# The pathophysiology of stress urinary incontinence: a systematic review and meta-analysis

**DOI:** 10.1007/s00192-020-04622-9

**Published:** 2021-01-08

**Authors:** Kobra Falah-Hassani, Joanna Reeves, Rahman Shiri, Duane Hickling, Linda McLean

**Affiliations:** 1 Faculty of Health Sciences, University of Ottawa, Ottawa, Canada; 2 Finnish Institute of Occupational Health, Helsinki, Finland; 3 Ottawa Hospital Research Institute, Ottawa, Canada; 4 Department of Surgery, Division of Urology, The Ottawa Hospital, Ottawa, Canada

**Keywords:** Stress urinary incontinence, Females, Ultrasound, Magnetic resonance imaging, Electromyography, Dynamometry

## Abstract

**Introduction and hypothesis:**

To evaluate the evidence for pathologies underlying stress urinary incontinence (SUI) in women.

**Methods:**

For the data sources, a structured search of the peer-reviewed literature (English language; 1960–April 2020) was conducted using predefined key terms in PubMed and Embase. Google Scholar was also searched. Peer-reviewed manuscripts that reported on anatomical, physiological or functional differences between females with signs and/or symptoms consistent with SUI and a concurrently recruited control group of continent females without any substantive urogynecological symptoms. Of 4629 publications screened, 84 met the inclusion criteria and were retained, among which 24 were included in meta-analyses.

**Results:**

Selection bias was moderate to high; < 25% of studies controlled for major confounding variables for SUI (e.g., age, BMI and parity). There was a lack of standardization of methods among studies, and several measurement issues were identified. Results were synthesized qualitatively, and, where possible, random-effects meta-analyses were conducted. Deficits in urethral and bladder neck structure and support, neuromuscular and mechanical function of the striated urethral sphincter (SUS) and levator ani muscles all appear to be associated with SUI. Meta-analyses showed that observed bladder neck dilation and lower functional urethral length, bladder neck support and maximum urethral closure pressures are strong characteristic signs of SUI.

**Conclusion:**

The pathology of SUI is multifactorial, with strong evidence pointing to bladder neck and urethral incompetence. While there is also evidence of impaired urethral support and levator ani function, standardized approaches to measurement are needed to generate higher levels of evidence.

## Introduction

Urinary incontinence has a prevalence of up to 28%, with stress urinary incontinence (SUI) being the most common form [1]. SUI is defined by involuntary loss of urine during tasks (e.g., coughing) in which bladder pressure exceeds the pressure at which the urethra has the capacity to remain closed [2]. Diagnosis of SUI and subsequent management decisions appear to be best evaluated by subjective report [3]; however, subjective measures do not offer any information about contributing pathology and do not inform any potential opportunity to personalize interventions.

Objective, summative measures of continence function, such as abdominal leak point pressure (ALPP) or Valsalva leak point pressure (VLPP), determined through urodynamic assessment, are used to confirm SUI, but do not reflect symptom severity or predict treatment outcome [4]. ALPP is defined as the intravesical pressure at which urine leakage occurs in the absence of a detrusor contraction. Yet there is currently no standard application of ALPP and no consensus on its utility in guiding management decisions [5]. While not validated through empirical data, McGuire et al. suggested that, among women with SUI, ALPP < 60 cmH_2_O is related to intrinsic sphincter deficiency while ALPP > 90 cmH_2_O is related to urethral hypermobility [6].

Maximum urethral closure pressure (MUCP) is another summative urodynamic measure used to confirm SUI. While MUCP does not capture true fluid pressure but rather an artifact of the total urethral closure force [7], and outcomes depend on the technology used (microtip catheter transducers, water perfusion catheters, air-charged balloon catheters [8]), it may have utility in terms of understanding the pathophysiology of SUI. Schick et al. suggested that MUCP is impacted by both urethral hypermobility [9] and intrinsic sphincter insufficiency [10], yet there are many continent women with low MUCP and there are women with SUI who demonstrate high MUCP [5]; as such, compensatory strategies, such as levator ani muscle (LAM) contraction, may also be important.

Prevailing theory suggests a combination of disruption in the supportive connective tissues of the bladder and urethra [11] and weakening of the muscular structures of the pelvic floor, bladder neck and urethral sphincters [12] all lead to reduced urethral closure pressure [13] and lower ALPP [5], functionally resulting in SUI [14, 15].

Support of the urethra depends on its fascial attachment to the arcus tendinous fasciae pelvis and connective tissue attachments to the pubis [16]. Impaired anatomical support of the bladder neck and proximal urethra is associated with urethral hypermobility, which is thought to impede the transfer of loads induced by the descending pelvic structures to the urethra, resulting in less extrinsic closure force, and ultimately with urine leakage. The mobility of the urethra can be detected visually [17] and through palpation and can be measured using magnetic resonance imaging (MRI) or ultrasound scanning (USS) [18]. However, detection may be biased when women are asked to perform tasks that may result in urine leakage; women may limit their effort [19] or co-contract the levator ani muscles (LAMs) [20] to avoid the embarrassment of leakage.

The pelvic floor muscles (PFMs), including the LAMs, coccygeus, perineal muscles, striated urethral sphincter (SUS) and external anal sphincter, form the base of the abdomino-pelvic cavity and contribute to the support of the pelvic contents and continence control [21]. The LAMs are considered a functional unit which provides support to the pelvic organs in the transverse plane (lifting) and compresses the urethra against the anterior vagina in the mid-sagittal plane (squeezing). Damage to or dysfunction of the LAMs is thought to be a contributor to SUI [22–25].

LAM structure and function can be evaluated through many different approaches (Table 1) [55, 56]. USS and MRI can identify gross damage to the LAMs such as avulsion [57], and strain (microtrauma) can be inferred through the size of the levator hiatus [58]. Manual palpation can be used to detect levator avulsion [59]; yet, while it is commonly used in clinic to assess LAM strength [60], there are limitations around reliability and precision [51]. Intravaginal dynamometry is recommended as the best approach to directly measure LAM force-generating capacity [61]; yet, as with manometry, only force contributions in a single plane are recorded, and measures may be contaminated by intra-abdominal pressure [48].

**Table 1:** Measurement and methodological issues associated used in the included studies

Assessment method	Measurement issue
Electromyography (EMG)	• Data susceptible to crosstalk, the recording of activity from nearby muscle groups that cannot be distinguished from true pelvic floor muscle activity • Valid comparisons not possible between groups without normalization because of differences in impedance, muscle depth and muscle fiber orientation • Normalization challenging because of possible difficulties in being able to perform voluntary maximum pelvic floor contractions • Normalizing PFM EMG data using MVCs reported to have the smallest standard error of measurement and minimal detectable difference compared to a cough, Valsalva and abdominal crunch maneuvers; however, the participants in the study did not have any form of PFM dysfunction and results may differ in a population with SUI [26] • Artifact from probe movement • Variety of devices used: Periform™ (NEEN Mobilis Healthcare Group, UK) [27–29]; Lifecare PR-02 (Everyway Medical Instruments Co., Taiwan) [30, 31]; VET-A (Nanjing Vishee Medical Technology, Ltd.) [32]; Femiscan™ (Mega Electronics Ltd., Kuopio, Finland) [33, 34]; STIMPON™ (Innocept Biobedded System GmbH) [35–39], a custom probe [25] and unspecified [40] as well as disposable surface electrodes (Mediwatch, UK or Medtronic, Minneapolis, MN) attached to a sponge [41, 42] • While the Periform™ and Femiscan™ have demonstrated poor between-day reliability [43], large coefficients of variation [44] and large standard error of measurement [29], to the authors’ knowledge, the reliability of the STIMPON, Lifecare PR-02 and VET-A probes have not been published in a peer-reviewed journal. The cylindrical tripolar electrode design of the STIMPON would be particularly prone to motion artifact, especially when used during dynamic tasks such as running and jumping [45] • Allegedly good reliability of PFM EMG variables in running across 10 steps in one session based on high ICCs; however, minimal detectable differences were very large (87% of MVC for maximum activity [46]), and high reliability does not reflect validity (i.e., crosstalk contamination), especially as high activity of hip external rotator muscles has been shown while running [47]
Ultrasound imaging (USI) and magnetic resonance imaging (MRI)	• Affected by bladder volume and the multiplanar orientation of the pelvic flor • Affected by posture • Lack of standardized procedures, positions, outcome measures and terminology
Manometry, dynamometry and perineometry	• Embarrassment about leakage might prevent women with SUI straining as directed, especially when assessors not blinded • Difficult to establish whether a true maximum was performed • Intra-abdominal pressure can be misinterpreted as forces having been generated through PFM action [48, 49] • Confounding effect of intra-abdominal pressure greater with perineometry (where air- or fluid-filled chambers inserted into the vagina record pressure changes within the chamber resulting from the sum of the forces acting on all surfaces of the chamber, including forces generated by the descent of the pelvic organs) than with dynamometry [50] • Measures of maximum force-generating capacity may be confounded by poor motor control [33]
Digital palpation	• Subjective and poor reliability [51] • Lacks the sensitivity to gauge small changes in pressure) [52]
Terminology	• Task nomenclature is not standardized • Straining sometimes referred to as during cough or Valsalva maneuver, yet not specified • Tendency for straining and Valsalva to be used interchangeably when they have been shown not to be equivalent [53]: The correct instructions suggested for a Valsalva have been described as: “take a breath, then close the mouth, pinch the nostrils with the thumb and the index finger, then blow air forcefully toward the blocked mouth and nostrils and direct the increasing pressure into the ears” while for straining: “take a breath, then contract the abdominal muscles and strain downwards with the intention to evacuate stool or urine” [53]. Using these instructions, with the Valsalva there was diaphragm and pelvic floor elevation, while with straining there was pelvic floor descent [53]. Another study showed that bladder neck displacement was similar in a cough and Valsalva in incontinent women, but was lower during the cough than Valsalva in both parous and nulliparous controls [54], despite greater abdominal pressures recorded during the cough than during the Valsalva across groups • Furthermore, it has also been shown that the Valsalva maneuver can be accompanied by co-contraction of the levator ani in nulliparous women, affecting measurement of bladder neck descent [20]

While electromyography (EMG) amplitude does not translate directly to force output [62], when applied correctly, kinesiological EMG recordings from the LAMs can be useful to determine the extent and timing of LAM activation during functional tasks, i.e., motor control. However, surface EMG recordings of the LAMs can carry a high risk to external validity and detection bias due to a number of measurement issues (Table 1), for example, concurrent activity recorded from nearby muscles is inseparable from that of the LAMs (crosstalk) [45]. Dynamic USS and MRI have identified urogenital landmark motion, through which LAM activation has been inferred [63] (e.g., anterior and cranial motion of the anorectal angle), an approach that has recently been validated [64].

Distinct from kinesiological EMG, clinical EMG involves the recording of evoked potentials and motor unit potentials, the latter normally studied using needle electrodes. Clinical EMG findings can be used to infer myopathic and neuropathic processes. While studies have been few, results have suggested that damage to the pelvic and/or pudendal nerves may be implicated in SUI [65]. While the external anal sphincter is part of the PFM complex, it is not thought to contribute to urinary continence function, but is an accessible muscle through which pudendal nerve integrity can be evaluated.

The SUS is considered part of the PFM complex, and it appears to be a major contributor to urinary continence control [66], along with the smooth muscle surrounding the urethra and bladder neck [67]. Contraction of the SUS, achieved through voluntary or automatic control, provides a direct closure force at the mid-urethra [68]. Additionally, intrinsic urethral closure forces are generated through longitudinal and circular smooth muscles of the urethral sphincter. Kinesiological EMG recorded from the SUS may provide valuable information about reflex and motor control; however, it is not commonly measured, as the sphincter muscles are not accessible without invasive methods. To supplement sphincteric closure forces, passive (bulk) forces are generated by perfusion of the urethral blood vessels and a hermetic seal is produced by mucoid secretions from the urethral epithelium [69].

In light of the complex interactions among tissue morphology, mechanical properties, perfusion, innervation and motor control, several factors may contribute to the pathophysiology of SUI, yet the evidence for many of these factors has not been systematically evaluated, and their relative importance is largely unknown. The aim of this systematic review and meta-analysis was to synthesize the evidence for the different morphological and pathophysiological mechanisms associated with SUI.

## Methods

### Search strategy

We used the Preferred Reporting Items for Systematic Reviews and Meta-Analyses (PRISMA) guideline [70] to develop the study protocol registered in PROSPERO (CRD42020180715). We conducted literature searches in PubMed (Table 2) and Embase from 1960 until April 2020 using predefined key terms. We limited the searches to adult females. We also searched Google Scholar and manually searched the reference lists of eligible articles for publications not identified in the initial search.

**Table 2:** PubMed search string

Search	Query	
#1	“Pelvic floor disorders”[Mesh] OR “pelvic floor”[Mesh] OR “pelvis”[Mesh] OR “pelvic floor muscle” OR “urethral mobility” OR “urethral support” OR “urethral closure pressure” OR “urethral sphincter” OR EMG [Text Word] OR “electromyography”[Mesh] OR electromyography [Text Word] OR “muscle contraction”[Mesh] OR “muscle contraction”[Text Word] OR “muscle function” OR “neuromuscular action” OR “ultrasonography”[Mesh] OR “diagnostic imaging”[Mesh] OR ultrasound [Text Word] OR “vaginal resting pressure” OR perineometry OR perineometer OR “oxford scale” OR ALPP[tiab] OR abdominal leak point pressure[tiab])	2,979,216
#2	“Urinary incontinence, stress”[Mesh] OR “urinary incontinence”[Mesh] OR “stress urinary incontinence” OR incontinent [Text Word]	35,419
#3	“Control groups”[Mesh] OR control [Text Word] OR continence [Text Word] OR continent [Text Word] OR “healthy volunteer” OR asymptomatic [Text Word] OR without stress incontinence [Text Word]	4,012,744
#4	Female [MeSH Terms] OR female [Text Word] OR women [MeSH Terms] OR women [Text Word] OR woman [Text Word]	8,929,022
#5	#1 AND #2 AND #3 AND #4	2842
Final	#5 NOT (Clinical Trial[ptyp] OR Review[ptyp])	2065/2091

### Study eligibility

Studies were deemed eligible if they: (1) were reported as full text in English; (2) reported the results of peer-reviewed research based on cross-sectional, case control or cohort studies on women with SUI; (3) included women > 16 years of age; (4) assessed some aspect of urogenital structure or function related to SUI; (5) concurrently recruited a comparison (control) group of continent women.

Studies were excluded from the review if: (1) the control group reported urogynecological symptoms including urgency incontinence, dyspareunia or pelvic organ prolapse, or were diagnosed with neurological disease/disorder or cancer; (2) in addition to SUI, cases had a concurrent urinary tract abnormality, e.g., fistula, tumor, etc.

### Data extraction

Data were extracted by three independent reviewers (KFH, JR and RS) including year of publication, country, study population, sample size, outcome measures, assessment method and adjustment for covariates.

### Quality assessment

The three reviewers (KFH, JR and RS) independently rated the risk of bias in the included studies using criteria adapted from the Effective Public Health Practice Project Quality Assessment Tool (Table 3) [71]. Studies were rated on selection bias (i.e., response rate and representativeness of the sample), detection bias (i.e., whether the outcome measures were valid and reliable) and confounding variables. Detection bias with respect to the diagnosis of SUI was not assessed because both self-report questionnaires and urodynamic methods have limitations, and there is still no gold standard for diagnosis [72]. Attrition bias was not considered applicable as studies were cross-sectional in nature. Lack of blinding was classified as performance bias (during data collection) and/or detection bias (during analysis) [73]. The authors discussed and resolved disagreements in quality ratings until consensus was reached including, when necessary, the input of the senior author (LM).

**Table 3:** Risk of bias assessment

Type of domain	Criteria definition	Classification (potential for bias)
Selection bias	Sampling method of the study population, representativeness (response rate, difference between responders and non-responders)	**Low:** Target population defined as representative of the general population or subgroup of the general population (specific age group, specific geographic area and specific occupational group) and response rate is 80% or higher Multicenter study **Moderate:** Target population defined as somewhat representative of the general population, a restricted subgroup of the general population, response rate 60%–79% Single-center study Limited details of the method of recruitment and study population **High:** Target population defined as self-referred/ volunteers, or response rate < 60% Single-center study Very limited details of method of recruitment and study population
Detection bias	Valid and reliable assessment of pelvic floor function	**Low:** Normalized EMG, ultrasound **Moderate:** Vaginal palpation, assessment method prone to some measurement issues **High:** EMG reported in μV, not normalized, assessment method prone to several measurement issues

### Data synthesis

The results were synthesized qualitatively, and, where possible, meta-analyses were conducted using a random-effects model in R Studio using the METAFOR package [74]. For studies that reported means and standard deviations for subgroups of patients with SUI, we calculated grand means and pooled standard deviations for total cases for meta-analysis. We used raw or standardized mean difference, the latter using Hedges’ g to weight group standard deviation by sample size, for continuous outcomes [75] and risk ratios for count data. We assessed heterogeneity across the studies using the I^2^ statistics [76].

## Results

**Fig. 1:**
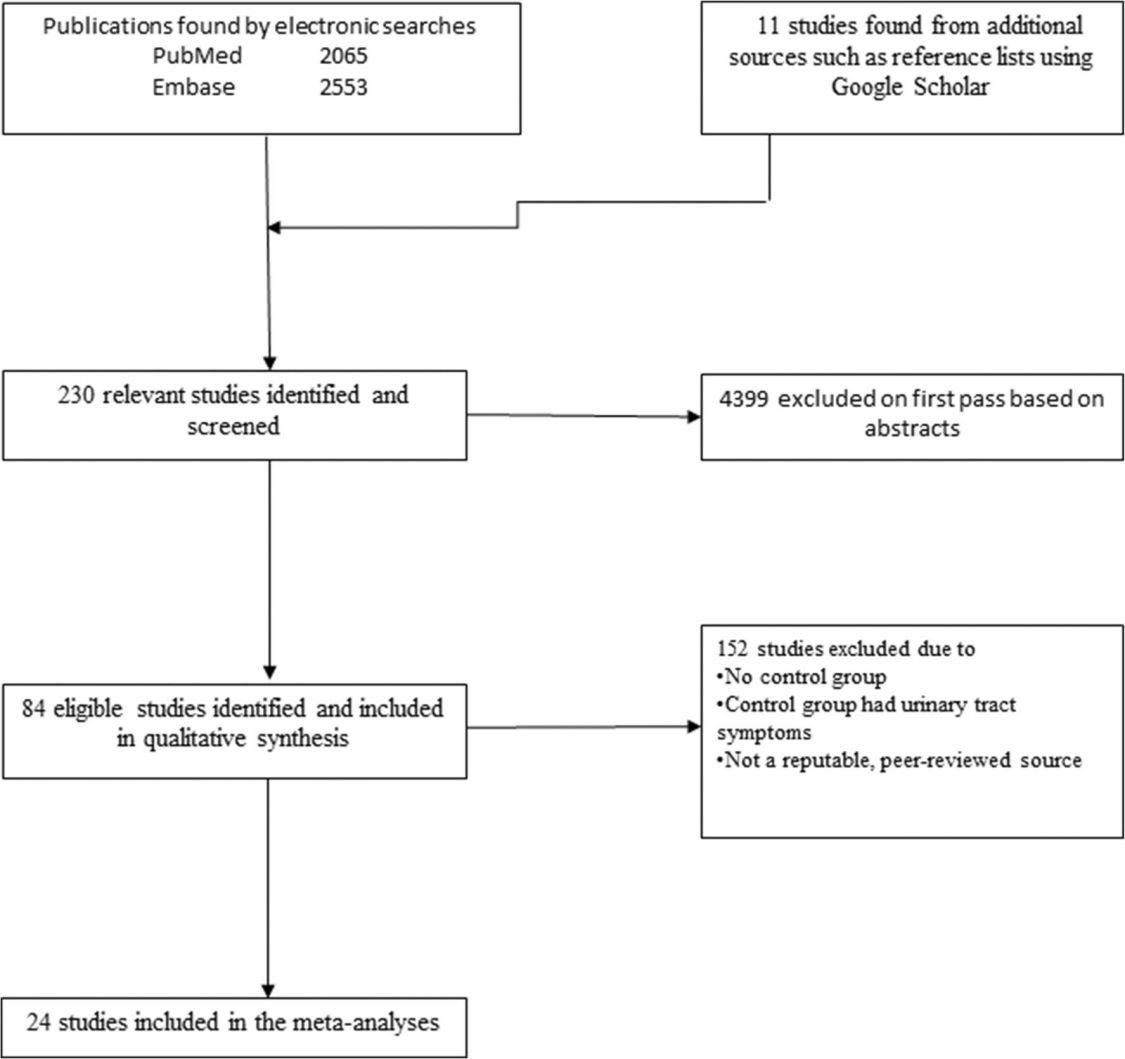
Flow chart of the search strategy and selection of studies

A total of 4629 abstracts were screened. Of those, 4399 abstracts were excluded on first pass, and 230 relevant studies were identified. Studies with no control group or with a control group that had other significant lower urinary tract symptoms were excluded (*n* = 150). No studies were excluded based on the age of the participants, and all reported on women aged ≥ 18 years. Two studies were excluded [77, 78] because they were considered to be from predatory journals [79]. Ultimately 84 studies were included in the review, and 24 studies were included in the meta-analyses (Fig. 1 and Appendix (Table 4). The majority of studies confirmed SUI through some form of self-report or urodynamics. Selection bias was rated as moderate or high in all cases. Eighteen studies [19, 27, 28, 32, 33, 66, 83, 88, 91, 93, 97, 108, 114, 130, 133, 135, 136] controlled for all or most major known confounders of urinary incontinence, 43 studies controlled for some confounders, and 23 studies did not match or control for any confounding factor. Over two thirds of the studies (61/84) did not report blinding the assessors to any of the outcomes. Detection bias ranged from low to high, with issues around measurement fidelity being associated with most assessment methods (Table 1).

## Summative measures of SUI

### Abdominal leak point pressure

No study using ALPP as an outcome met the inclusion criteria.

### Functional urethral closure

Several studies found that maximal urethral pressure (MUP) measured at rest [14, 83, 96–98, 115, 122] and MUCP (the difference between MUP and bladder pressure using filling cystometry) [10, 14, 15, 93, 96–98, 105, 115, 116] were lower in stress-incontinent than in continent women. Urethral closure pressure measured in supine position was negatively associated with greater severity of SUI [95]. Among urethral closure pressure, measures of urethral support and other parameters (e.g., levator hiatus size, urethral axis on LAM contraction, LAM strength, levator defect status) and maximal cough (intravesical) pressure, MUCP was the strongest determinant of SUI (*n* = 211, Cohen’s d = 1.47 [93]). In other studies, the best predictor of clinically significant SUI was urethral incompetence, defined using residual MUCP, closure pressure measured during a cough [10] or MUCP alone [93]. Minimum urethral closure pressure on coughing was also significantly lower in stress-incontinent women than in continent controls [98], whereby women were instructed to cough forcefully until leakage was observed at the urethral meatus [10].

**Fig. 2:**
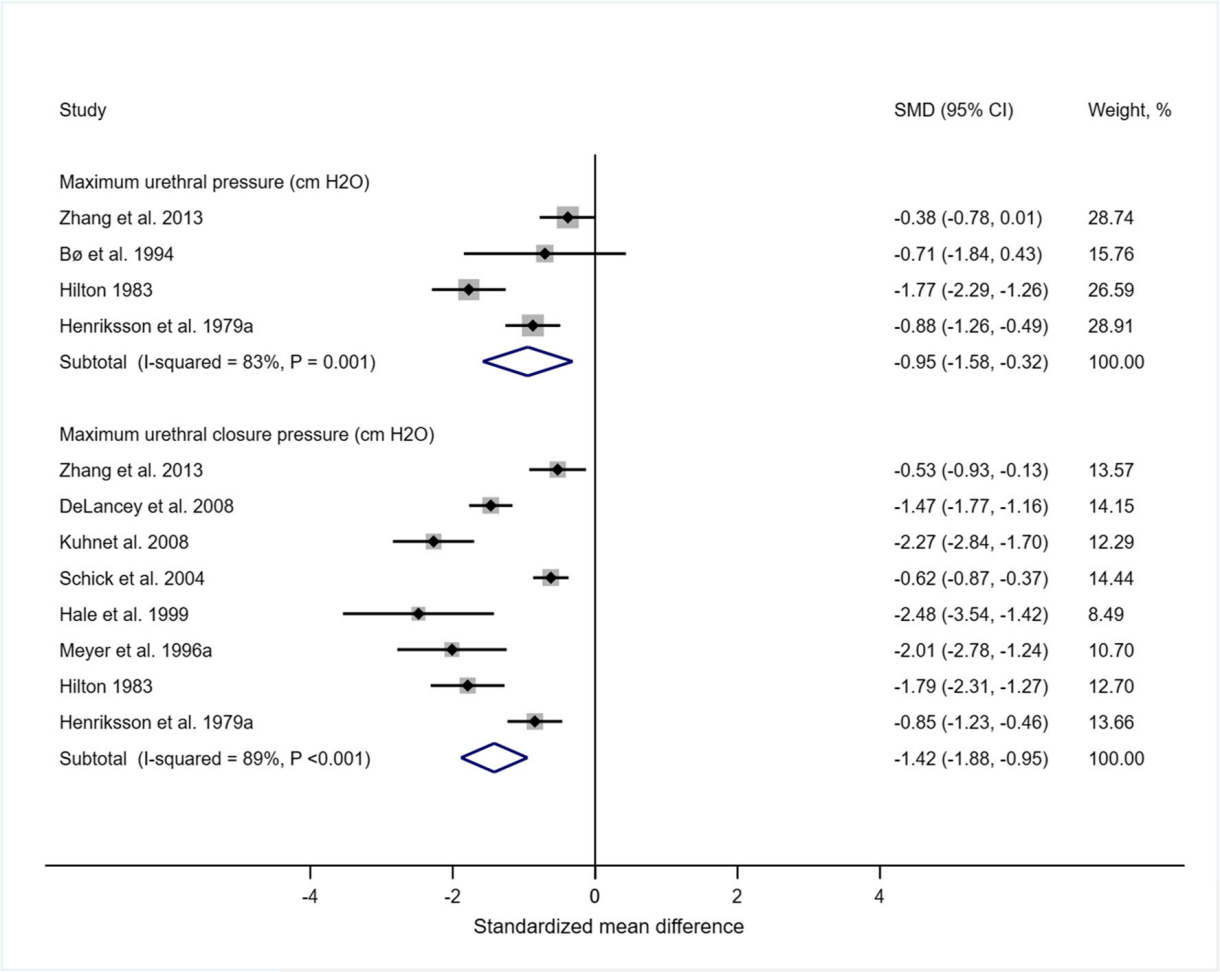
Standardized mean differences (SMD) in the maximal urethral pressure (MUP) and maximal urethral closure pressure (MUCP) in stress-incontinent women compared with continent women. For studies by Hilton (1983) and Henriksson et al. (1979b), average values across SUI severity and age groups were used respectively. For the study by Meyer et al. (1996a) an average of SUI groups with and without a low pressure urethra (failure to generate MUCP > 20 cmH_2_O) in supine position was used. For the study by Hale et al. (1999) the MUCP at rest was used. For the study by Kuhn et al. (2008) estimates were made from the published figure (SUI group: 23 ± 13.5 cmH_2_O; control group: 55 ± 20 cmH_2_O)

The meta-analyses of standardized mean difference showed that MUP (the inward pressure exerted by the walls of the urethra) (Hedges’ g = −0.95, 95% CI -1.58, −0.32, 4 studies, Fig. 2) was lower in stress-incontinent (*n* = 1034) compared to continent (*n* = 336) women, and MUCP (Hedges’ g = −1.42, 95% CI -1.93, −0.92, 8 studies, Fig. 2) was lower in stress-incontinent (*n* = 1122) compared with continent (*n* = 342) women. Similarly, MUP (pooled raw mean difference = −16.39 cm H_2_O, 95% CI -5.98 to −26.80, 4 studies) and MUCP (pooled raw mean difference = − 26.52 cm H_2_O, 95% CI -35.63 to - 17.41, 8 studies) were significantly lower in women with SUI compared to continent women.

## Individual factors contributing to SUI

### Urethra and bladder neck

#### Morphology

##### Urethral morphology

Nine studies reported on urethral morphology, six using USS [88, 97, 102, 104, 106, 136] and three using MRI [101, 110, 111]. Compared to continent women, the thickness of the mid-urethra in women with SUI seen on USS was not significantly different in the lateral aspects of the striated muscle layer [104], the longitudinal and circular smooth muscle layers [104] or more generally [106]. However, using USS, cohorts of stress-incontinent women have demonstrated shorter and more cranial positioning of the urethral sphincter complex at rest [88] and smaller area and circumference of the sphincter muscles compared to continent controls [102], the latter consistent with MRI findings that stress-incontinent women had significantly thinner SUSs than continent women [101]. Indeed, MRI is the better measurement for this application due to tissue orientation and boundary conditions that can interfere with tissue resolution on USS [137].

Through transvaginal USS, it was found that women with SUI had a distinct midurethral echogenicity pattern where the loose urethral structures were hypoechoic and the anterior region of the mid-urethra was more echogenic compared to controls after adjustment for confounders [120]. Consistent with this, using shear wave elastography, women with SUI were found to have lower stiffness of the SUS than continent women [136], yet boundary conditions may be an issue with this USS approach.

##### Urethral length

Total urethral length was reported using urethrocystometry [96], microtransducer catheter [98], sonography [119] and MRI [111], and results are inconsistent. Authors reported both shorter [96, 98, 111], longer [119] and no difference [123] in urethral length between stress-incontinent and continent women.

**Fig. 3:**
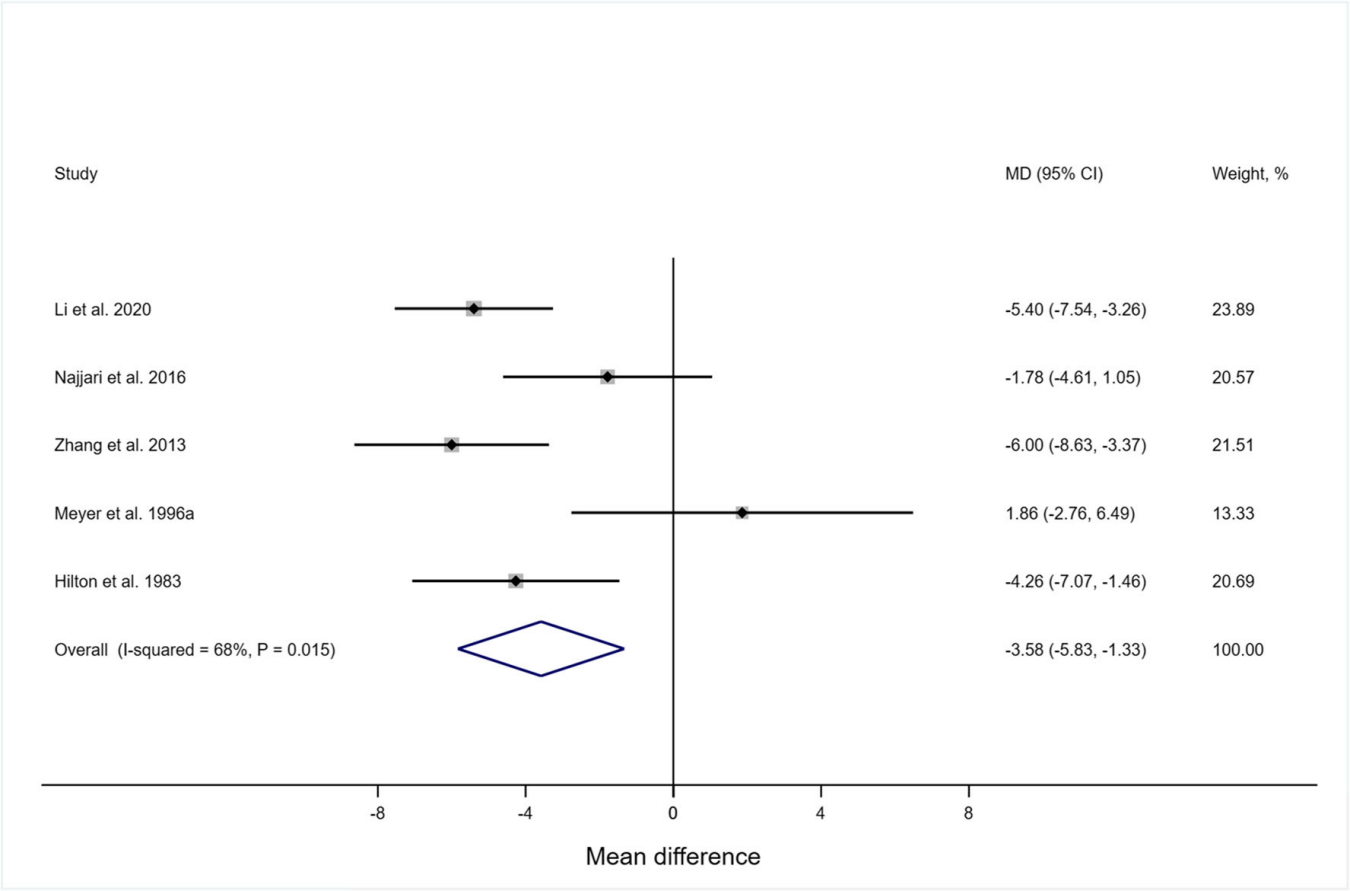
Mean difference (MD) in functional urethral length in stress-incontinent women compared with continent women. For the study by Li et al. (2020), the perimenopausal control group was used in this analysis

Functional urethral length, defined as the length of the urethra along which urethral pressure exceeds intravesical pressure, presumably represents the location of the urethral sphincters. Several studies [14, 96–98, 111, 119], but not all [116] found greater functional urethral length in continent than in stress-incontinent women. Shorter functional urethral length appears to be associated with greater severity of SUI [95, 98]. Meta-analysis showed that functional urethral length was 3.58 mm (95% CI −5.83 to −1.33 mm, 5 studies) shorter in stress-incontinent women (*n* = 521) compared with continent women (*n* = 109, Fig. 3).

##### Urethral perfusion

Using 3D color-Doppler transvaginal USS, fewer periurethral vessels and less periurethral blood flow were found in stress-incontinent than in continent women [112].

##### Bladder neck funneling

**Fig. 4:**
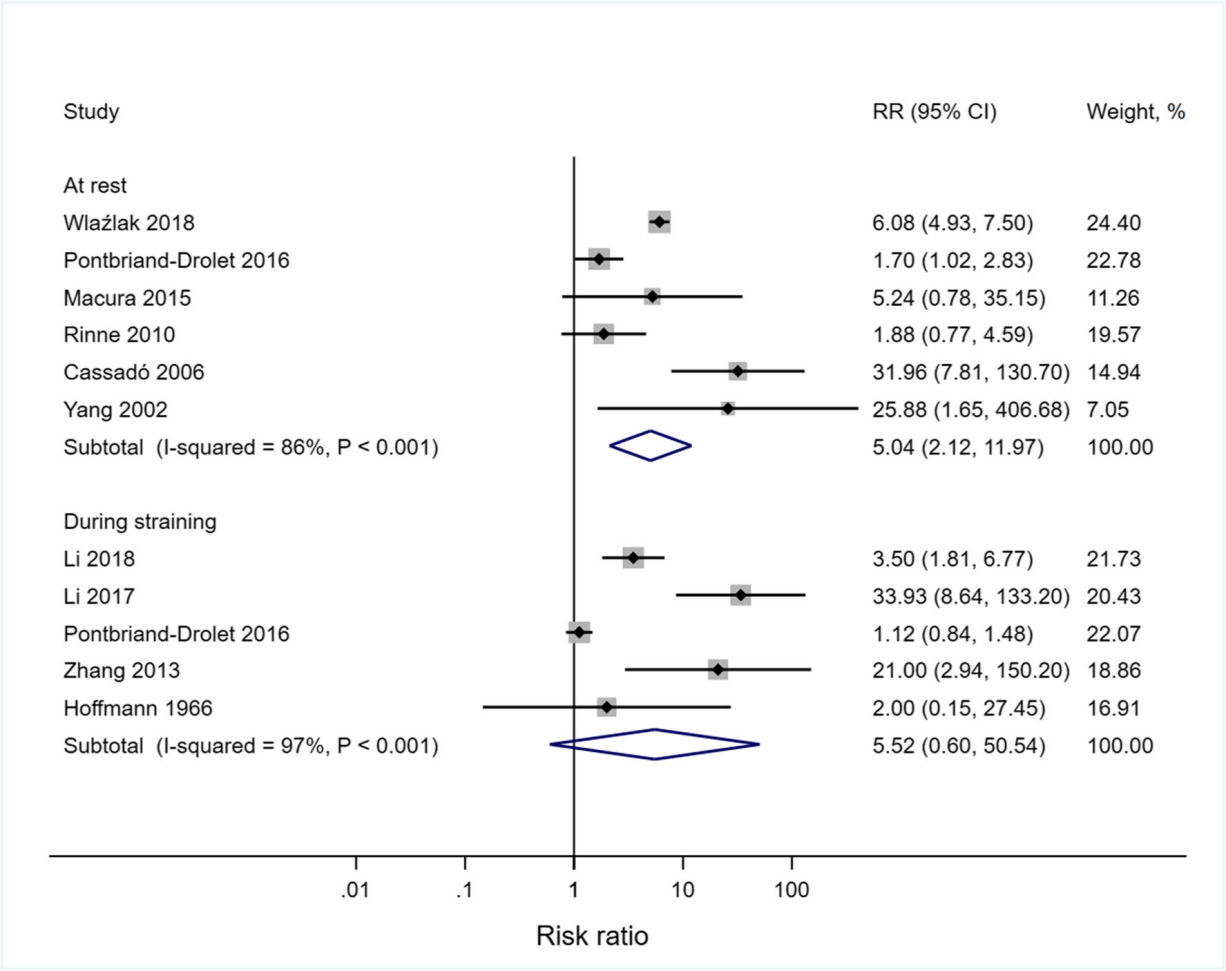
Risk ratio for bladder neck funneling in stress-incontinent women compared with continent women. For the study by Hoffmann and Ulrich (1966), the SUI group without pelvic organ prolapse was used in this analysis

Bladder neck funneling (or dilation), measured using USS, MRI or cystourethrography, is described as an observed funnel-shaped opening of the proximal urethra [138], measured dichotomously as present or absent. Bladder neck funneling was studied with women at rest in six studies [19, 87, 114, 123, 133, 134], during straining in five studies [14, 19, 99, 109, 110] and during defecation in one study [111]. A meta-analysis of six studies suggests that funneling is five-fold (risk ratio = 5.04, 95% CI 2.12–11.97 at rest and risk ratio = 5.52, 95% CI 0.60–50.54 during straining) more prevalent among stress-incontinent (*n* = 1195 and *n* = 181 for rest and straining, respectively) than continent women (*n* = 775 and *n* = 193 for rest and straining, respectively, Fig. 4). However, most studies did not control for confounding factors such as age, parity and body weight (Fig. 4). In another study, bladder neck incompetence, defined as a widely separated lumen, was found in 42/60 stress-incontinent women, but in none of 14 continent women [106].

#### Urethral neurophysiology

Among stress-incontinent women, responsiveness to urethral electrostimulation was lower [91], urethroanal reflex latency was more prolonged [91], neural conduction velocity in the perineal branch of the pudendal nerve (which innervates the SUS) was significantly slowed [126], and dorsal nerve and urethral mucosal electrosensitivity was diminished [129] relative to control groups. However, there were no differences in pudendal nerve terminal motor latency between women with and without SUI in another study [91]. In a larger study (*n* = 286), signs of neuropathic changes in the urethral sphincter, in the form of longer motor unit potential duration and pudendal nerve terminal motor latencies [116], were observed among women with SUI. Conversely, shorter urethral sphincter motor unit potential duration was found in a stress-incontinent group compared to a continent group [66], and, during bladder filling, the women with SUI demonstrated smaller motor unit potential amplitudes, lower numbers of turns per second and lower turns/amplitude ratios in the SUS [66].

#### Urethral support

##### Para-urethral connective tissues

Dense connective tissue arises primarily from the vagina and periurethral tissues and attaches to the pelvic wall laterally at the arcus tendineus fasciae pelvis and to the medial edge of the LAM.^1^ Lesions of the urethral support structures seen on MRI [101] were more prevalent in stress-incontinent than in continent women [110, 111, 127]. For instance, in one study, defects in the periurethral ligament were found in 76% of stress-incontinent women and in 32% of parous controls (*p* < 0.001, *n* = 58) [111]. While greater pubovaginal distance and periurethral ligament disruption were significantly associated with SUI, in a multivariable model, only periurethral ligament disruption was significantly more common in incontinent than in continent women [114]. Among 31 middle-aged women, periurethral ligament symmetry reduced the odds of incontinence by 87% [114]. The urethropelvic ligaments were significantly thinner in stress-incontinent women [106], but the length of the pubourethral “ligaments” was similar between women with and without SUI (*n* = 74) [106].

##### Bladder neck position

Bladder neck position was reported in several studies using USS [54, 87, 90, 113, 117, 132]. Compared to their continent counterparts, women with SUI demonstrated shorter distances between the bladder neck and the lower margin of the symphysis pubis at rest and during straining [90] and shorter distances from the bladder neck to the central axis of the symphysis pubis in standing, but not in supine position [54, 117]. In women with SUI, a larger distance from the symphysis pubis to the urethra at rest in supine position [87] was observed, and the bladder neck tended to sit in a more posterior and caudal position [87, 132] compared to continent women. Another study showed no difference in the resting position of the bladder neck in the horizontal or vertical plane between continent women and those with SUI [113]. It was not possible to conduct a meta-analysis for bladder neck position because of the variation in measurement approaches (e.g., using the central axis of the symphysis pubis [54, 90, 117] vs. a line through the apex [132] and measuring the direct [90, 132] vs. perpendicular distance from the bladder neck to symphysis pubis [54, 117]).

##### Urethral angular orientation

**Fig. 5:**
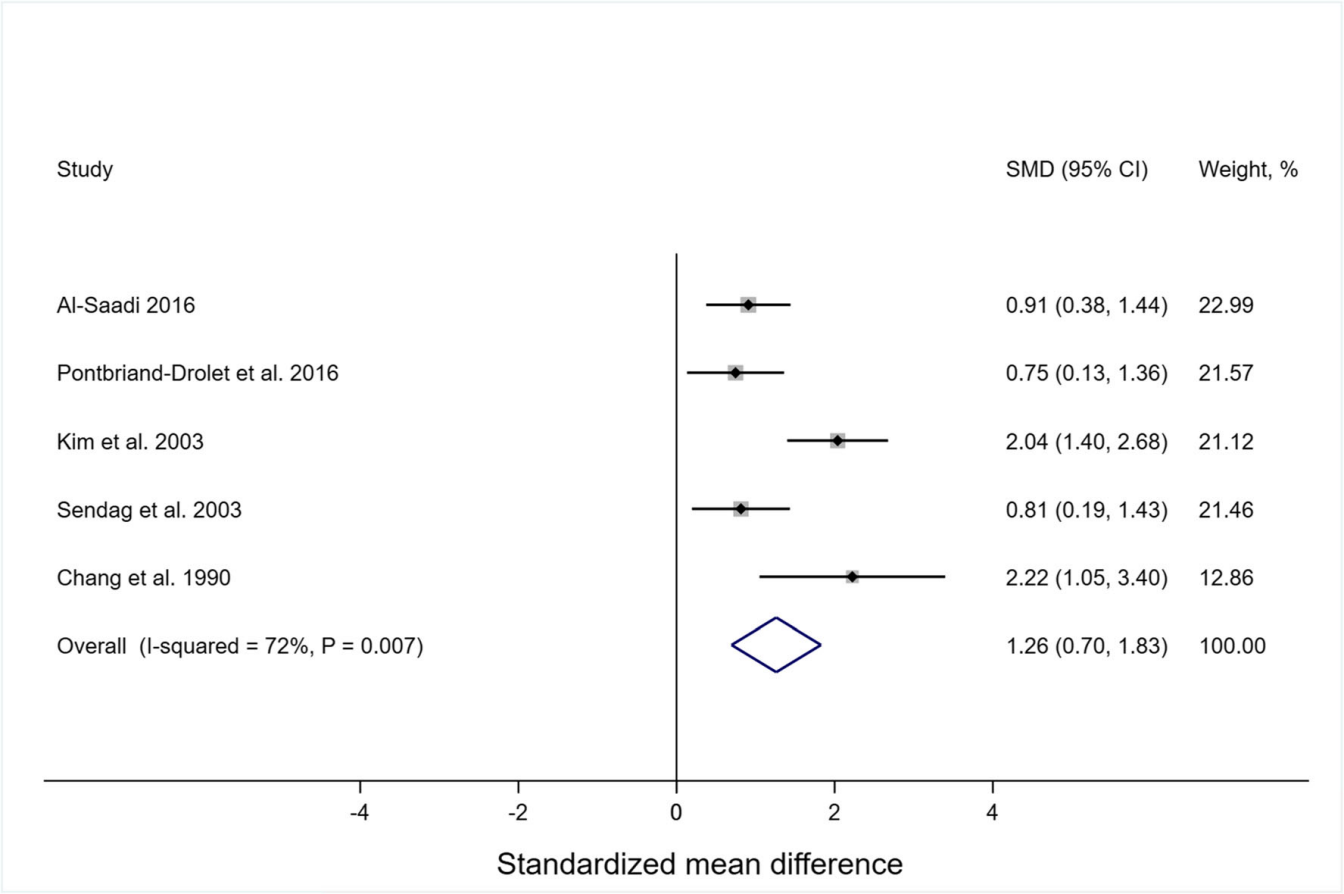
Standardized mean differences (SMD) of beta angles measured at rest in stress-incontinent women compared with continent women

Angular orientation of the urethra has been measured using MRI [19, 101] and USS [14, 19, 81, 89, 109, 125, 134, 136] employing varying definitions. Compared to continent women, women with SUI had larger rotation (α) angles at rest and during straining, defined as the angle between the axis of the proximal urethra and central axis of the symphysis pubis [81, 134], the angle between a line drawn through the bladder neck parallel to the probe and a line through the apex of the pubic bone [132], or the proximal urethral rotation angle, with a lack of clarity regarding the task [109]. However, when defined as the angle between the vertical axis and the urethral axis (a.k.a. the urethral axis angle), there was no difference in α angles at rest between women with and without SUI [110, 125], and, while during Valsalva there was no difference in one study [110], there were larger α angles in a stress-incontinent group in another study [125]. The β angle (also referred to as the posterior urethrovesical angle, posterior vesicourethral angle or retrovesical angle) was measured as the intersection between lines drawn along the urethra and the bladder base [19, 81, 89, 101, 109, 125, 136], the angle from the bladder base to the symphysis pubis [14], the angle from the bladder neck to the vaginal wall [132] or undefined [99, 109]. Regardless of definition, women with SUI typically had larger β angles at rest than continent women [14, 19, 81, 89, 101, 109, 125, 132, 136], with the exception of one study [99]. Meta-analyses showed (Fig. 5) there was a large effect of incontinence on the β angle [standardized mean difference: Hedges’ g = 1.26, 95% CI 0.70, 1.83, 5 studies; weighted mean difference = 19.2° (95% CI 13.3° to 25.2°, 5 studies)], with larger β angles observed among stress incontinent (*n* = 159) than continent (*n* = 92) women. Urethral mobility was assessed clinically using a cotton swab inserted into the distal urethra (Q-tip test) while women remained supine and at rest [93]. Although this test has limited accuracy [140], women with SUI demonstrated larger angular deviations of the cotton swab from horizontal [93] compared to those without SUI. Other findings include that, compared to continent women, women with SUI demonstrated a larger retropubic space [101] and a larger bladder neck-symphyseal angle at rest [134], but iliococcygeal angles and levator plate angles [110] did not differ.

#### Kinematics

##### Urethral mobility

The mid-urethra was observed through USS to move more caudally during straining in women with SUI compared to continent women [123], and the proximal urethra was also more mobile during coughing [97]. Concurrent with urethral sphincter incompetence, prolapse of the urethra into the anterior vaginal wall during the Valsalva maneuver was more common in women with than without SUI [10].

##### Bladder neck mobility: i. angular rotation

Compared to continent women, women with SUI demonstrated larger rotation angles of the urethrovesical junction, yet definitions varied among studies^2^ [14, 87, 90]. In a multivariable model, bladder neck rotation during straining (referred to as sliding^3^) had the highest sensitivity (92%) and specificity (80%) to diagnose SUI among 383 women [87]. Larger bladder neck–symphyseal angles were described for women with SUI versus continent women during maximal Valsalva maneuver in another study, but differences between the angle at rest and during Valsalva were not reported [134]. In other studies, there were no differences between women with and without SUI when in angular rotation of the bladder neck was measured during straining [132] or during when the rotation of the long axis of the urethra was measured during a Valsalva maneuver [136].

##### ii. Linear translation

Studies found larger bladder neck linear descent during Valsalva maneuver and/or coughing [54, 90, 109, 117, 136] and straining [82], shorter distance between the bladder neck and the lower margin of the symphysis pubis during straining [90] and more bladder neck mobility in the craniocaudal direction during coughing [132] in stress-incontinent women compared with their continent counterparts. Using transrectal USS, stress-incontinent women demonstrated > 1 cm of caudal motion of their urethrovesical junction during straining, while continent women had < 1 cm of caudal motion during straining [82]. However, other studies showed no differences between incontinent and continent women in the extent of displacement or descent of the bladder neck during coughing [113, 124] or depression of the base of the bladder during straining [99]. It was not possible to conduct a meta-analysis for bladder neck displacement because of the differences in conditions during which it was measured across studies (e.g., during coughing [113, 132], Valsalva [109, 136] or straining (either cough or Valsalva) [87, 90] and a lack of reporting of means and standard deviations for each group [53, 82, 124].

### Levator ani

#### Morphology

Stress-incontinent women had less skeletal muscle content, fewer muscle fibers in each LAM fascicle [15] and higher connective tissue content [15, 23] in their LAMs than were observed in continent women. Asymmetry of the puborectalis muscles was more common in stress incontinent women in one study [101], and, while there were no differences between women with and without SUI in terms of overall levator defects (asymmetry, hypertrophy or disruption) in a subsequent study [111], a significantly greater percentage of pubococcygeal muscle defects was found in stress incontinent women (45–66%) than in their continent counterparts (10–28%) [111, 127]. The thickness of the LAM measured by MRI [100, 127] and USS [121] was significantly lower in women with SUI compared with continent women [100, 121, 127]. MRI showed degeneration of the LAM in 45% of women with SUI [23]. In continent women, a sharp dorsally angulated levator sling was seen in transverse MRI sections [23], yet this angulation was lost in 66% of stress-incontinent women [23].

#### Neurophysiology

There were no differences in the presence of caudal-dorsal motion of the clitoris [135] or cranial-ventral movement of the anorectal junction toward the symphysis pubis during coughing between women with and without SUI [135]. Such motion presumably reflects automatic or reflex contraction of the LAMs and/or perineal muscles. In the external anal sphincter, stress-incontinent women had significantly prolonged motor unit potential duration [129], higher tonic activation amplitudes and higher mean numbers of turns/s [80] as well as higher estimates of mean fiber density [80]. However, during maximum voluntary contraction (MVC) there was no difference between groups in the mean amplitude of the EMG interference pattern in the external anal sphincter [80]. There were also no significant differences in the density or mean amplitude of the interference pattern in the puborectalis muscles when women with and without SUI were compared [80].

#### Passive tissue resistance

Shear strength of the anterior vaginal wall was significantly lower in stress-incontinent women than in continent women [103], yet using intravaginal dynamometry, the peak resistance to passively stretching the LAMs and paravaginal tissues did not differ between continent and stress-incontinent women [130].

#### Active force generation

##### Strength

Stress-incontinent women had weaker LAMs on assessment by palpation using manual muscle testing [24] and other subjective rating [131]. Compared to continent women, women with SUI generated lower intravaginal pressures on maximal effort PFM contraction when measured using perineometry [24] and generated lower forces using intravaginal dynamometry [22, 130]. The pressures/forces measured at the posterior vaginal wall in stress-incontinent women were significantly lower than in continent women both at rest [60, 84] and during maximal effort PFM contraction [60]. Additionally, force was generated more slowly in the women with SUI [60, 85], and they were less able to sustain force [84]. Similarly, stress-incontinent women had lower LAM endurance than continent women in one study [24], but not in another [86]. Conversely, among athletes, LAM strength was greater in those with SUI than in those without [94], yet in runners who ran ≥ 20 km a week, there was no significant difference in LAM strength between the continent and incontinent groups [92].

In other studies there were no significant differences between continent and incontinent women in terms of intravaginal pressure/closure force generated during maximal effort LAM contraction when measured using air-filled balloons connected to pressure transducers mounted on the anterior and posterior aspect of an intravaginal probe [34], a microtip sensor [83], unspecified manometry [86] or a multisensor device (MLA-P1, Pliance_ System; Novel; Munich, Germany) [84]. Yet external anal sphincter contraction measured using manometry was weaker in stress-incontinent women than in continent women [129].

Using transperineal USS during maximal effort PFM contraction, one study showed greater elevation of the proximal urethra in incontinent women versus continent controls [93], while another showed the opposite [24]. In one study, the mid-urethra rose higher in women without than with SUI when performing a PFM contraction [123], but in another study there was no difference in bladder neck elevation between groups performing the same task [41]. There was no between-group difference in the change in anorectal angle (lines drawn along the posterior walls of the anus and rectum) from rest to maximal PFM contraction [19].

These conflicting results, as well as the detection bias associated with LAM strength measurement and an overall lack of assessor blinding in studies comparing LAM strength between women with and without SUI, lead us to conclude that there is limited evidence for LAM strength impairment in SUI.

##### Motor control

Of the 16 studies that recorded EMG amplitude [27–37, 40, 41, 80, 108, 118], only 5 normalized EMG amplitude to generate valid comparisons between cases and controls [28, 35–37, 118].

##### Tonic activation of the LAMs

Three studies reported that EMG tonic activity (activity recorded at rest) of the LAMs was lower in stress-incontinent women than in continent women when they assumed sitting [27], standing [108], lying [108] and unspecified [32] postures. In another study, tonic EMG activity in the LAMs was not different between stress-incontinent and continent women in an unspecified posture, both before and after a fatigue test [40]. However, EMG amplitude was not normalized in any of these studies, putting them at high risk of detection bias [141, 142]. Baseline/resting EMG amplitude of the LAM was similar or lower in women with SUI versus continent women in three studies where statistical comparisons between the groups were not performed because of the nature of the studies (i.e, two training studies that did not compare between groups at baseline [30, 31] and a reliability study that recruited separate groups of women with and without SUI [29].

##### Phasic activation of the LAMs

In some studies women with SUI were reported to have lower EMG activity of the LAMs during contractions [25, 34, 108] and MVCs [29] versus continent women, although the last study did not make between-group comparisons [29]. Conversely, in other studies there was higher LAM EMG activity in women with SUI during contraction [32], a static hold (although statistical comparisons were not performed) [31] and both prior to and during the postural response associated with unexpected loading [28]. The severity of incontinence experienced by women created a differential effect on LAM EMG activity—women with mild SUI demonstrated higher EMG activation amplitudes than controls (*p* < 0.05 and *p* = 0.07 for baseline and response, respectively), while women with more severe SUI demonstrated no significant difference in EMG amplitudes than controls [28]. During coughing, there was no effect of SUI or its severity on LAM EMG activity [33]. In another study, maximum EMG amplitude during LAM contraction was inversely related to SUI severity [34]; however, the risk of detection bias was high: EMG signals were not normalized and there was no way of determining whether participants achieved their true maximum activation.

Other studies reported no group differences in EMG activity of the LAM during fast voluntary contractions [35], MVCs [33, 35] or submaximal contractions [41]. Similarly, no differences in EMG variables, normalized to LAM MVC, were found between women with and without SUI during jumping [38] or running [36]. Longer relaxation times during repeated fast voluntary contractions were found in women with SUI versus controls, despite no differences in peak EMG amplitudes between groups [37].

In continent women, the superficial perineal muscles always contracted before the deep PFMs in six positions, while in stress-incontinent women the reverse sequence was observed in three of these positions [42]. No difference was found in the power spectra of intravaginal EMG during treadmill running between stress incontinent women and continent women [39].

### Other

While change in intra-abdominal pressure measured during Valsalva or cough was not different between stress-incontinent and continent women [124], incontinent women generated higher intravesical pressures during cough and Valsalva [93]. In stress-incontinent women, compared to continent women, greater anterior pelvic tilt was found from digitized photographs [108] and greater vertical displacement of a marker on the fifth lumbar vertebrae during running, without any other differences in kinematics [92]. There were no differences in clitoral sensory threshold (measured as the intensity at which the woman was first able to perceive an electrical stimulus) between stress-incontinent and continent women [91].

## Discussion

Urodynamic measures such as ALPP and MUCP describe the summative result of the many factors that contribute to urinary continence. Indeed, meta-analysis suggests that low MUCP [93] is strongly associated with SUI, and, while low ALPP also appears to reflect the presence of SUI, no studies were found that compared ALPP between women with SUI and a control group of women with no urogynecological disorders. An appropriately controlled study comparing ALPP between women with and without SUI, matched on known confounders (e.g., age, parity, BMI, smoking, activity level) is needed.

While few studies have simultaneously explored multiple factors that contribute to SUI [10, 93], the outcome of this review suggests that SUI is indeed multifactorial [143], with evidence pointing to deficits in urethral and bladder neck structure and support, neuromuscular, vascular and mechanical impairment of the SUS, and defects in but not weakness of the LAMs. Meta-analyses showed that bladder neck dilation at rest and shorter functional urethral length are strong characteristic signs of SUI. Insufficient data were available for meta-analyses related to LAM structure or function.

Among the studies included in this review, few controlled for confounding factors associated with SUI such as age, parity, obesity and menopause. The relative volume of the SUS and blood vessels decreases with age [144] and menopause [145], and this may coincide with an increased prevalence of SUI [19]. The risk of SUI also increases with higher parity [146], obesity [147] and a history of moderate/heavy smoking [148]. Many of the included studies described control groups of continent women who were younger, had fewer children and had lower BMIs than women with SUI and did not report on smoking history. Cumulative loading of the pelvic floor through athletic activities may also be an important confounder [149]. Indeed, observed associations between some of the urethral and bladder neck characteristics and SUI may be attributable to differences between cases and controls in terms of demographic or behavioral risk profiles. Lastly, despite evidence of differences in the prevalence and presentation of SUI by race and ethnicity [150], few studies reported on race/ethnicity [66, 93, 131], whereby only one matched [66] and one adjusted the statistical model based on race [93], however the latter sample was mainly (> 94%) Caucasian.

The studies in this review measured outcome variables during several different tasks including coughing [113, 132], Valsalva [109, 136] and straining (sometimes described as either during a cough or Valsalva) [87, 90]. Although there was a tendency to use the terms straining and Valsalva (forced expiration or bearing down) interchangeably, these have been shown not to be equivalent (Table 1) [53].

There was high variation in the instruments and outcomes used to measure similar phenomena, and several measurement issues could bias results (Table 1). For example, urethral and bladder neck position and kinematics were measured using the Q-tip test [93, 116], palpation [24], USS [54, 88, 97, 102, 104, 106, 117, 136] and MRI [101, 110, 111], with many different landmarks and measurement strategies, and differences were found across studies in terms of posture, bladder volume and task.

The presence of SUI itself was often assessed both with self-report questionnaires and urodynamically; however, both methods have limitations. There is no gold standard for diagnosis [72]. While self-report measures and a detailed pelvic floor examination are likely the best way to direct treatment and measure success [3], objective measures such as ALPP and MUCP may be more useful when studying SUI pathophysiology. However, it is well known that different instrumentation and approaches result in different urethral pressure measurements obtained during urodynamics [151]. Furthermore, the lack of blinding in many studies may have led to biased findings, as assessors may vary their instructions during data collection or analyze results differently if they are aware of patient diagnosis. There is a clear need to develop standard terminology [152] protocols and measurements to allow for comparisons among studies and to ultimately improve our understanding of SUI.

Impairments in urethral and bladder neck structure and support, evidenced through ultrasound imaging and MRI, emerged as being strongly associated with SUI in women. Damage to the periurethral, paraurethral and pubo-urethral connective tissues [101] may occur during pregnancy, labor and delivery, through chronic coughing or other repetitive loading, or with obesity and may impact the position of the urethra and bladder neck at rest as well as its mobility during tasks that challenge continence [101, 114]. The wide variation in how these measures are performed is problematic and requires standardization to be useful in clinical investigations.

Bladder neck dilation (or funneling) emerged through meta-analysis as being highly prevalent in women with SUI (risk ratio = 5.23 at rest and 4.99 during straining). Bladder neck dilation may develop through damage to or denervation of the smooth muscle sphincter around the proximal urethra [124] as well as to the proximal aspects of the longitudinal and circular smooth muscles of the urethra. While bladder neck dilation is observed in 50% of continent women [124], urinary continence appears to be maintained in those women through the SUS [124] and perhaps the LAMs. As with the limitations of imaging noted above, standardized methods for the quantification of bladder neck funneling are currently lacking.

Studies included in this review suggest that there is neurophysiological evidence of denervation injury to the SUS in women with SUI [66, 128]. While a lower turns-amplitude ratio [66] is a non-specific finding [153], increased pudendal nerve terminal motor latency to the striated sphincter [116], longer urethral sphincter motor unit potential durations and fewer turns per second [66] are all suggestive of axonal damage with subsequent re-innervation through axonal sprouting [154]. In a separate multivariate model, however, motor unit potentials recorded from the SUS during bladder filling showed shorter durations, lower amplitudes and larger numbers of phases in stress-incontinent women versus continent women, which may reflect myopathic changes. As such, there may be different presentations that lead to sphincter incompetence. Regardless, neurophysiological findings at the SUS are consistent with morphological findings of reduced cross-sectional area in women with SUI. While confirmatory studies are needed, both Heessakkers et al. [155] and Kenton et al. [66] have suggested a role for routine clinical EMG examination in the evaluation of urethral sphincter insufficiency. Myopathic or neurpathic defects in the urethral sphincter may indeed be important predictors of surgical failure [156].

While during MVC there was no difference between women with and without SUI in the amplitude of the EMG interference pattern in the external anal sphincter [80], or in the density or mean amplitude of the interference pattern in the puborectalis muscles [80], motor unit loss needs to be severe [157] before any decrease in EMG amplitude is evident; therefore, these results are inconclusive.

Far more information is available about the role of urethral structure, support and function in SUI than the role of the LAMs. Studies included in this review suggest that the LAMs in women with SUI are more likely to have lesions [110, 111, 127], have fewer muscle fibers [15] and more connective tissue [15, 23], have reduced muscle bulk [100, 121, 127] and show evidence of degeneration [23]. These findings are consistent with palpation, dynamometric and imaging findings that suggest that women with SUI sometimes have reduced force generating capacity [22, 130, 131] and sometimes reduced endurance [24, 84] of their LAMs; yet the results are inconclusive, and insufficient data were available for meta-analysis. While there is strong evidence for PFM training as an intervention for SUI [158], the mechanism through which PFM training improves continence symptoms remains unknown and may be compensatory through improved motor control and/or through the concurrent hypertrophy of the SUS [159].

The interpretation of EMG findings from the included studies was particularly limited by issues related to the acquisition and analysis of data. Most recording devices have demonstrated poor between-day (test-retest) reliability [43]. None of the studies that reported on PFM EMG activation during functional activities presented convincing data to rule out the presence of crosstalk or motion artifact contamination. Less than half of the studies reported on normalized EMG amplitudes, which has been identified as a shortcoming in PFM research [160]. Admittedly standardizing a reference contraction of the LAM may be challenging [27, 141, 159], but normalization is required to draw valid conclusions [141].

While mild-to-moderate physical activity may have a protective effect on the PFMs and may decrease the risk of developing SUI, high impact activities may increase the risk of developing SUI [149]. Indeed, in one study, a moderate negative correlation was found between activity level and vaginal resting pressure in the stress incontinent group (r = −0.46 (*p* = 0.04), whereas a weak positive correlation was found in the control group r = 0.38 (*p* = 0.02) [86], suggesting different pathological mechanisms between athletes and non-athletes. The four studies comparing LAM activation between women with and without SUI during running [36, 39, 92, 107] were inconclusive, yet data were highly susceptible to detection bias and running durations may not have been long enough [161] to induce observable changes (8 min [92] or unspecified [36, 39]). Given that running/jogging is the most common high-impact activity to cause leakage [149], this is an important area for future research.

There appears to be much redundancy built onto the urinary continence mechanism in women. Defects in one aspect of continence control, for example, damage to the proximal urethra, may be compensated by another, for example, the SUS. This redundancy suggests that multiple failures may be needed before symptoms of SUI emerge. These multiple failures may occur because of a single event (for example, vaginal delivery) or may emerge after subsequent exposures (deliveries, chronic coughing, high BMI or physical activity, smoking) or with advancing age or the onset of menopause. Sequential insults may explain epidemiological data whereby SUI prevalence rises with age until after the childbearing years, then rises again after around age 70 years [1], often presenting as mixed incontinence in older women [72].

Large cross-sectional studies with concurrent evaluation of morphology, neurophysiology, vascularity and function are needed to understand the relative importance of and the interactions among the different factors associated with SUI. Longitudinal studies are also needed to understand the cause(s) and progression of leakage while considering the impact of age and/or exposure to risk factors. Once we are better able to measure pathological processes, targeted interventions based on predominant underlying pathophysiology could be tested in clinical trials.

## Appendix

**Table 4:** Studies included in the review

Study authors and country	Participants	Outcome measures	Assessment method	Risk of bias		Blinding	Summary results	Adjustment for covariates and additional comments
				Selection	Detection			
Aanestad and Flink (1999) [80] Sweden	SUI: N = 24 (*N* = 11 for pressure recordings) and age = 52 (32–81) years. Controls: *N* = 7 and age = 32 (18–52) years. SUI confirmation not reported	Inference pattern: number of turns per second and mean signal amplitude (μV); fiber density in puborectal and external anal sphincter muscles, cytometry and pressure profiles	EMG of pubo rectal and external anal sphincter muscle with empty urinary bladder at rest and during MVC as well as during cystometry. Millar double tip microtransducer for pressure profiles	High	Low	No	Compared to controls SUI group had greater mean number of turns/s (43.2 vs. 25.2, *p* < 0.01) and amplitude at rest (233.1 μV vs. 206.4 μV, *p* < 0.01) in external anal sphincter. No significant differences between groups in external anal sphincter during MVC or in puborectalus at rest or during MVC. Mean fiber density of external anal sphincter greater in SUI group than controls (2.01 vs. 1.33, *p* < 0.01)	Unadjusted. Greater percentage of parous women in SUI group than control group. BMI not reported. SUI group older than controls
Al-Saadi (2016) [81] Iraq	SUI: N = 30 and age = 37.5 ± 12.5 years. Controls: *N* = 30 and age = 35.3 ± 10.2 years. SUI was confirmed urodynamically, but method not described	The angle between the axis of the proximal urethra and the central axis of the symphysis pubis (α angle), and the angle between the proximal urethra and the posterior vesical wall (β angle)	Transperineal ultrasonography supine at rest and on straining (Valsalva maneuver). Bladder filled to around 150 ml	High	Low	No	Significantly larger α angle (*p* < 0.001) in SUI than in controls both at rest (64.4° ± 12.8° vs. 43.9° ± 1.5°) and on straining, (83.8° ± 14.2° vs. 54.4° ± 2.6°). Significantly larger β angle (*p* < 0.001) in SUI group than in controls both at rest (125.3° ± 18.7° vs. 107.5° ± 19.8°) and on straining (153.6° ± 26.9° vs. 123.9° ± 22.7°)	Unadjusted. No significant difference in age between groups. BMI and parity not reported
Ballmer et al. (2020) [35] Switzerland	SUI: *N* = 22; age = 45.9 ± 9.7 years and BMI = 21.6 ± 2.0 kg/m^2^. Controls: *N* = 28; age = 38.9 ± 10.3 years and BMI = 21.7 ± 1.8 kg/m^2.^ Secondary analysis of Leiter et al. (2017) and Moser et al. (2018) studies, whereby SUI was confirmed with ICIQ-UI SF and personal history	Cross correlation, SPM, peak amplitude (%MVC) during fast voluntary contraction and timing of peak amplitude in fast voluntary contraction and MVC of PFM	Surface EMG in upright standing with electrodes in true differential and faux differential configurations. MVC scored with Oxford grading scale, symmetry determined by palpation	High	Low	No	No significant differences in EMG variables between groups. Half of SUI group had asymmetry of PFM strength, vs. 4/28 of controls. Of a total of 62 comparisons of EMG parameters of MVC and FVC, only one comparison showed significant differences between the two configurations (4th peak in fast voluntary contraction *p* = 0.015)	Unadjusted. SUI group significantly older. BMI similar between groups. Parity not reported
Bergman et al. (1988) [82] USA	SUI: *N* = 44 and mean age = 53 years. Controls: *N* = 24 and mean age = 29 years. Total age range = 21–78 years. SUI confirmed with clinical examination and urodynamics	Urethrovesical junction height drop during straining	2D B-mode transrectal ultrasound at rest and maximal straining. Bladder filling not reported	High	Low	Yes	86% of SUI had a drop in urethrovesical junction of > 1 cm during straining (mean: 1.3 ± 0.6 cm), while 92% of controls had a drop < 1 cm during straining (mean: 0.54 ± 0.29 cm)	Unadjusted. SUI group older and greater parity than controls. BMI not reported. Also did a Q-tip test but had worse sensitivity and specificity than ultrasound
Bø et al. (1994) [83] Norway	SUI: *N* = 7; age = 19.9 ± 1.9 years and BMI = 21.4 ± 1.3 kg/m^2^. Controls: *N* = 6; age = 20.4 ± 2.3 years and BMI = 21.1 ± 1.8 kg/m^2^. SUI determined through self-report on a questionnaire	PFM strength, PFM and urethral sphincter EMG activity (qualitative), striated urethral sphincter muscle EMG with simultaneous urethral, bladder-pressure	Urodynamic measures, pressures with mircotip catheter, needle EMG, strength measured with palpation	High	Low	Yes	6/7 in SUI group and 1/6 controls had urethral sphincter incompetence as determined on urodynamic evaluation. No other significant differences between groups	Matched for age and types of sports usually performed. No differences in age, BMI, physical activity between two groups and all were nulliparous
Burti et al. (2015) [40] Brazil	SUI: N = 30; age = 53.63 ± 11.30 years and BMI = 27.95 ± 4.57 kg/m^2^. Controls: *N* = 26; age = 50.73 ± 8.94 years and BMI = 24.80 ± 4.88 kg/m^2^. SUI determined using ICIQ-UI- SF	EMG variables at baseline and after an endurance test: amplitude (μV) at rest before and after contraction and during sustained contraction reaching maximum voluntary; median frequency, time to peak contraction, time to fatigue and heart rate	Surface EMG with intravaginal probe	High	High	No	Similar (non-normalized) EMG amplitudes between groups. During the endurance task, incontinent women reported PFM fatigue earlier than continent women	Unadjusted. Age did not significantly differ between SUI group and controls, but SUI group had higher BMI, reported more vaginal deliveries and fewer cesarean section than controls
Cacciari et al. (2020) [84] Brazil	SUI: N = 23; age = 48.2 (44.7–51.7) years and BMI = 27.5 (25.9–29.1) kg/m^2^. Controls: *N* = 31; age = 35.3 (31.5–38.9) years and BMI = 23.4 (21.8–25.0) kg/m^2^. SUI confirmed with King’s Health Questionnaire	Peak pressure, pressure-time integral, time for peak pressure to drop to 75% and 50%	Spatiotemporal distribution of pressures along the vaginal cavity assessed with a cylindrical multisensory device (MLA-P1, Pliance® System; novel; Munich, Germany) assessed during rest, maximum and sustained PFM contractions	Moderate	Low	No	Peak pressure at rest mean [95% CI] lower in the SUI group (6.7 [5.7–7.7] kPa) than controls (9.0 [7.7–10.3] kPa, *p* = 0.01). Peak pressure during maximum contraction not significantly different between groups (*p* = 0.39). During sustained contraction pressure-time integral mean [95% CI] lower in t SUI group (214.3 [166.3–262.4] kPa·s) than controls (295.3 [254.9–335.7] kPa·s, *p* = 0.01; mean [95% CI] time for pressure to drop to 75% of peak shorter in SUI group (1.7 [1.2–2.2] s) than controls (3.0 [2.3–3.8] s, *p* = 0.005) and to drop to 50% of peak was shorter in SUI group (4.6 [3.8–5.4] s) than controls (7.1 [6.4–79] s, *p* < 0.001)	SUI group older, had a greater BMI and greater parity than controls (*p* < 0.01)
Carafini et al. (2019) [85] Brazil	SUI: N = 24; age = 48.2 ± 8.1 years and BMI = 27.5 ± 3.6 kg/m^2^. Controls: N = 24; age = 35.3 ± 10 years and BMI = 23.4 ± 4.2 kg/m^2^. SUI confirmed with King’s Health Questionnaire	Features of intravaginal pressure that have greatest discrimination capability between SUI group and controls	Pressure recorded along vaginal cavity assessed with a cylindrical multisensory device (MLA-P1, Pliance® System; novel; Munich, Germany) with a 10 × 10 array of sensors assessed during maneuvers: maximum contraction, Valsalva, endurance and wave	High	Low	No	Six extracted frequencies were best able to distinguish between SUI and control group. Peak pressure at posterior medial sensors during wave maneuver significantly less in SUI vs. controls (*p* < 0.05). Time to peak pressure in Valsalva of the right deep sensors significantly greater in SUI vs. controls (*p* < 0.05)	SUI group older and had greater BMI, but significance not tested. Parity not reported. Third division of sensor had three lines, each with a 10-sensor perimeter surrounding the cylinder: cranial (corresponding to first three lines of sensors from vaginal opening), medial (four mid-lines of sensors), and caudal (three last lines of sensors)
Carvalhais et al. (2018) [86] Portugal	SUI: *N* = 20; median ± IQR age = 21.0 ± 15 years. Controls: *N* = 38; median ± IQR age = 24.0 ± 7.0 years. SUI confirmed with ICIQ-UI SF	Vaginal resting pressure, PFM strength and PFM endurance and relationship to physical activity, (MET–min/wk) calculated using the formula min. of an activity per day × the numbers of days per week × the specific MET score for each activity	Manometry in standing using Peritron Perineometer 9300 (LABORIE Medical Technologies Canada ULC.) Physical activity assessed with IPAQ short form. MET–min/wk. calculated using formula min. of an activity per day × the no. of days per week × specific MET score for each activity	High	Low	No	Vaginal resting pressure and PFM strength and endurance not significantly different between groups. Correlation between vaginal resting pressure and MET-min/week in SUI group: moderate and negative (r = −0.46 (*p* = 0.04); in controls weak and positive r = 0.38 (*p* = 0.02)	No significant differences between groups except SUI group classified as having a high PA level compared to controls (65.0% vs. 34.2%, respectively; *p* = 0.030)
Cassadó et al. (2006) [87] Spain	SUI: *N* = 138 and age = 54 ± 13 years. Controls: *N* = 245 and age = 35 ± 13 years. SUI confirmed with urodynamics	Urethral anatomical length, urethral sphincter, distance from symphysis pubis to urethra at rest, distance from crossing point of symphysis axis with urethra to bladder neck, distance from symphysis to bladder neck, and presence of funneling. Sliding described as difference between urethra-bladder neck distance measured at rest vs. on straining (greater of Valsalva or cough)	B-mode ultrasonography with bladder filled to approximately 175 ml (75–275 ml)	Moderate	Low	Yes	In a multivariable model, sliding was higher in SUI than in controls. Sliding had highest sensitivity (92%) and specificity (80%) to diagnose SUI. Distance from symphysis pubis to urethra at rest was higher in SUI group than in controls (10.89 ± 3.05 mm vs. 7.95 ± 2.40 mm, *p* < 0.001). Distance from crossing point of the symphysis axis with urethra to bladder neck at rest was lower in SUI group than in controls (20.58 ± 4.77 mm vs. 26.55 ± 4.25 mm, *p* < 0.001)	Adjusted for age, parity and menopause. BMI not reported
Cassadó Garriga et al. (2017) [88] Spain	SUI: *N* = 95; age = 50.7 ± 13.4 years and BMI = 25.73 ± 4.86 kg/m^2.^ Controls: *N* = 78; age = 52.6 ± 14.3 years and BMI = 26.51 ± 5.25 kg/m^2^. SUI determined using ICIQ-UI SF	Urethral mobility and bladder neck descent	2D and 3D-4D translabial ultrasound, supine. Urethral mobility assessed by measuring bladder neck descent on Valsalva maneuver. Bladder empty	Moderate	Low	Yes	The length of the urethral sphincter complex at rest was significantly shorter in SUI group (3.15 ± 0.43 mm) than controls (3.54 ± 0.38, *p* < 0.001). Urethral mobility was significantly higher in SUI group than in control group (21.85 ± 8.3 mm vs. 12.82 ± 6.8 mm, *p* < 0.001)	Association of length of the urethral sphincter complex with SUI adjusted for urethral mobility. Groups did not significantly differ in age, BMI, parity, cesarean section, operative vaginal delivery, cystocele, rectocele, or vaginal vault prolapse. Uterine prolapse more common in control group and levator ani avulsion more common in SUI group
Chamochumbi et al. (2012) [22] Brazil	SUI: *N* = 16; age = 48 ± 7 years and BMI = 26 ± 3 kg/m^2^. Controls: N = 16; age = 37 ± 8 years and BMI = 37 ± 8 kg/m^2^. SUI defined through subjective report and the absence of detrusor over activity on urodynamic testing	Evaluation of PFM strength (active forces generated in N), passive force and vaginal cavity aperture (mm) through passively stretching the PFMs	A custom intravaginal instrumented speculum (dynamometer) to measure forces exerted in four directions – anterior, posterior, and lateral during maximum effort PFM contraction	High	Low	Yes	Women with SUI had significantly lower anteroposterior active force compared to continent women (0.1 ± 0.1 N vs. 0.3 ± 0.2 N, *p* < 0.01). No significant differences in left-right active force, (*p* = 0.2), passive force (*p* = 0.89) or vaginal cavity aperture (*p* = 0.06)	Unadjusted. SUI group were older and had higher BMI than controls. Parity not reported
Chang et al. (1990) [89] China	SUI: *N* = 14 and age = 37–67 years. Controls: N = 7 and age = 31–62 years. SUI confirmed with history, physical examination and urodynamics	Posterior urethrovesical angle (PUVA)	Transrectal sonographic cystourethrography, radiographic chain cystourethrogram under strain and non-strain	High	Low	No	From USI: PUVA larger in SUI group than controls at rest (142.9° ± 6.7° vs. 125.7° ± 8.8°) and during straining (168.2° ± 9.3° vs. 128.5° ± 8.1°)	Unadjusted. BMI and parity not reported. Similar age range between groups
Chen et al. (1997) [90] Taiwan	SUI: *N* = 37 and 35.6 (24–46) years. Controls: *N* = 65 and age = 37.5 (23–49) years. SUI was determined urodynamically	Distance between bladder neck and lower margin of symphysis pubis at rest and during straining (Valsalva or cough), rotational angle of bladder neck, distance between the bladder neck and an arbitrary line at rest and during straining, and descent of bladder neck	2D transperineal B-mode ultrasound at rest and during Valsalva, supine. Bladder volume comfortable (150–250 ml)	High	Low	No	In SUI group shorter distance between bladder neck and lower margin of symphysis pubis at rest (26.8 ± 4.0 mm vs. 29.3 ± 3.3 mm, *p* < 0.01) and during straining (19.4 ± 3.5 mm vs. 23.0 ± 3.8 mm, *p* < 0.01) compared to controls. Greater rotation angle in SUI group than controls (42.9° ± 19.7° vs. 17.6° ± 14.9°, *p* < 0.01). Distance from bladder neck to arbitrary line less in SUI group than in controls at rest (18.2 ± 5.6 mm vs. 21.3 ± 5.5 mm, *p* < 0.01) and during straining (0.6 ± 6.8 mm vs. 12.3 ± 8.5 mm, *p* < 0.01). Greater bladder neck descent in SUI group than in controls (17.6 ± 7.3 mm vs. 9.0 ± 6.9 mm, *p* < 0.01)	Adjusted for parity. BMI not reported. Groups of similar age
de Aguiar Cavalcanti et al. (2013) [91] Brazil	SUI: *N* = 33. Less severe SUI: *N* = 14 and age = 52.6 ± 15.0 years. More severe SUI: *N* = 19 and age = 56.7 ± 11.5 years. Controls: N = 19; age = 47.3 ± 9.9 years and SUI confirmed urodynamically	Abdominal leak point pressure, pudendal nerve terminal motor latency, pudendal somatosensory evoked potential latencies, urethral and clitoral sensory thresholds and urethroanal reflex latency	Urodynamic studies performed using Laborie Aquarius system. Electrophysiological tests performed using a Neuropack Sigma system, including an isolated nerve/muscle stimulator	Moderate	Low	No	No differences in clitoral sensory threshold, pudendal somatosensory evoked potential latency, or pudendal nerve terminal motor latency between the SUI group and controls. Longer urethroanal reflex latencies in the SUI group compared to the controls (*p* < 0.05)	Adjusted for age, height, parity, and number of vaginal deliveries by the propensity score method. SUI group older than control group
de Melo Silva et al. (2020) [92] Brazil	SUI: *N* = 11; age = 41.9 ± 11.6 years and BMI = 22.1 ± 2.5 kg/m^2^. Controls: *N* = 17; age = 38.5 ± 7.3 years and BMI = 22.4 ± 2.1 kg/m^2^. Women who ran at least 20 km per week, had been running for ≥ 6 months. SUI confirmed with ICIQ-UI SF	Vertical displacement of a marker on 5th lumbar vertebrae (cm), knee flexion during load response, foot strike. Strength (Oxford Scale), Endurance (s), Manometry (mmHg) and pad test (g)	8-min run at 75% of max. Kinematics from video at 60 Hz. PFM function assessed with palpation and vaginal squeeze (Peritron™(Cardio Design, Oakleigh, VIC, Australia))	High	Moderate	No	Greater vertical displacement in SUI group controls 13.0 ± 2.0 cm vs. 11.0 ± 2.0 cm (*p* = 0.02). No other significant differences in outcome variables between groups and no associations between PFM function and running kinematics	SUI group had greater running experience (*p* = 0.06) and weekly distance (*p* < 0.01). No significant differences in age, BMI or parity
DeLancey et al. (2008) [93] USA	SUI: *N* = 103; age = 47.7 ± 9.3 years and BMI = 30.4 ± 6.6 kg/m^2^. Controls: *N* = 108; age = probable typo in manuscript and BMI = 27.6 ± 5.6 kg/m^2^. SUI confirmed with voiding diary and clinical examination	Urethral support, urethral mobility (urethral axis inclination with respect to the horizontal), urethral closure force, and levator ani muscle strength. Regression to predict SUI	Urethral support was assessed by the Pelvic Organ Prolapse Quantification (POP-Q) System. Urethral mobility measured through a cotton- swab test. Bladder pressure and urethral sphincter closure force assessed urodynamically by urethral profilometry. Levator ani muscle strength assessed using an instrumented vaginal speculum	Moderate	Low	Yes	Maximal urethral closure pressure was 42% lower in SUI group than in controls (40.8 ± 17.1 vs. 70.2 ± 22.4 cm H_2_O, d = 1.47, *p* < 0.001). SUI group had greater urethral axis of inclination at rest (−0.8° ± 11.8° vs. −6.3° ± 15.1°, *p* = 0.004). Urethro-vaginal point (point Aa) descended more in SUI group than in controls on POP-Q (−0.6 ± 0.8 cm vs. –1.0 ± 0.8 cm, *p* < 0.001). SUI group generated higher intravesical pressures during cough vs. controls (143.2 ± 43.4 cm H_2_O vs. 126.4 ± 34.3 cm H_2_O, *p* = 0.002). Maximal urethral closure pressure best predictor of SUI. Prevalence of levator defects did not differ between groups (*p* = 0.31)	Matched for age, race, parity and hysterectomy status. No difference in menopausal status, hormonal therapy or medical conditions between groups. Regression adjusted for BMI
Devreese et al. (2007) [42] Belgium	SUI: *N* = 50; age = 52.8 ± 8.0 years. Controls: *N* = 32; age = 52.4 ± 9.7 years. BMI < 25 kg/m^2^ in both groups. SUI confirmed with 24-h pad test and leakage verified during or shortly after a cough or Valsalva after drinking 500 ml of water	The timing of the onset of contraction of the superficial relative to the deep PFMs during a maximum voluntary contraction of the PFMs	Perineal and intravaginal surface EMG using surface electrodes and a vaginal sponge (Medtronic) in six different positions: supine knees flexed, supine knees straight, sitting leaning forward, sitting upright, standing leaning forward, standing upright. Onsets calculated with threshold method	Moderate	Low	No	In the continent women the superficial muscles always contracted before the deep muscles in all 6 positions, while in the incontinent group the reverse sequence was observed in 3 of 6 positions (supine position with knees straight, the sitting while leaning forward position and the standing while leaning forward position)	Unadjusted. age and parity did not differ between groups. BMI not reported
Dornowski et al. (2018) [30] Poland	SUI: *N* = 22. Controls: *N* = 42. Total age = 23 ± 3 years and BMI = 22 ± 3 kg/m^2^. Future sport professionals. SUI confirmed with questionnaire	EMG amplitude (μV)	Surface EMG using Lifecare probe during a quick flick, static contraction and baseline/rest before a training programme. Unclear if standing or supine	High	High	No	No differences between groups	Unadjusted. age and BMI in individual groups not specified. Parity not reported. Not clear how different types of incontinence were considered
Dornowski et al. (2018) [31] Poland	SUI: *N* = 37 and mean age = 30.2. Controls: *N* = 76 (2 groups); mean age = 30.2 years. Pregnant women, mean 21 weeks. SUI confirmed with Incontinence Impact Questionnaire (IIQ)	EMG amplitude (μV)	Surface EMG using Lifecare probe during a quick flick, static contraction and baseline/rest before a training programme. Unclear if standing or supine	High	High	No	Mean baseline activity of SUI group: 3.26 μV, control group 1: 2.88 μV and control group 2: 3.05 μV	Unadjusted. Age and BMI in individual groups not specified. Not clear how different types of incontinence were considered
dos Santos et al. (2019) [94] Brazil	SUI: *N* = 21; age = 24.0 ± 4.9 years and BMI = 22.6 ± 3.2 kg/m^2^. Controls: *N* = 19; age = 24.3 ± 5.0 years and BMI = 22.3 ± 2.4 kg/m^2^. Nulliparous professional female athletes who competed at the municipal level SUI confirmed with ICIQ-UI SF	Modified Oxford scale, PFM endurance?, perineometer output (cmH_2_0), trunk: peak torque (Nm); peak torque (% body weight); work and power	PFM function and strength assessed using modified Oxford Scale and a perineometer. Abdominal muscle function and strength assessed using a 4-Pro isokinetic dynamometer	Moderate	Moderate	Yes	PFM measured with perineometer significantly greater in SUI group (33.9 ± 10.1 cmH2O) vs. controls (25.4 ± 12.8 cmH2O, *p* = 0.028). No other significant differences between groups. Positive association between PFM and abdominal muscle strength in both groups	Unadjusted. Age and BMI not significantly different between groups. Both groups nulliparous
Frauscher et al. (1998) [95] Austria	SUI: *N* = 21. Controls: *N* = 11. Total age range: 22–76 years. SUI severity determined with Ingelmann/Sundberg classification (grades I–III)	Sphincter muscle thickness and change in distance from sphincter- transducer between rest and contraction (ΔSTD) to serve as a measure of contractility of the muscle	Intraurethral ultrasound (supine), cytometry and urethral profilometry	High	Low	No	Sphincter muscle thickness: Controls = 3.5 ± 0.4 mm; grade I SUI = 3.4 ± 0.6 mm; grade II SUI = 3.3 ± 0.7 mm; grade III SUI = 1.7 ± 0.4 mm, *p* < 0.001. ΔSTD: Controls = 1.8 ± 0.05 mm; grade I SUI = 1.7 ± 0.06 mm; grade II SUI = 1.0 ± 0.04 mm; grade III SUI = 0.1 ± 0.01 mm	Unadjusted. BMI, parity and group age ranges not reported. Additional variables recorded without results presented for controls and/or mixed results for an incontinence group that included other types of incontinence
Gunnarsson and Mattiasson (1999) [25] Sweden	SUI: *N* = 58. Controls: *N* = 173. Total age range: 20–76 years. SUI confirmed urodynamically	Maximum EMG amplitude (μV)	EMG recorded with a custom probe supine during repeated short two second squeeze contractions	High	High	No	Significantly less non-normalized maximum EMG amplitude during squeeze contraction in those in SUI group over 50 years of age (*n* = 29) compared to controls (*p* < 0.001)	Matched by age and parity. BMI not reported
Hale et al. (1999) [15] USA	SUI: N = 10 (*n* = 3 for biopsies); age = 64.8 ± 11.1 years and BMI = 28.2 ± 3.6 kg/m^2^. Controls: N = 17 (*n* = 11 for biopsies); age = 40.6 ± 3.4 years and BMI = 24.9 ± 6.1 kg/m^2^. SUI defined based on International Continence Society definition	Maximum urethral closure pressure (MUCP), urethral sphincter EMG: fibrillation potentials; number of motor units found; duration; polyphasia (%), amplitude (μV) and muscle fiber content	Urodynamic tests, urethral sphincter needle EMG and urethral biopsy	High	Low	Yes	MUCP at rest and at full bladder capacity significantly lower in SUI group than controls (30.4 ± 15.5 cmH_2_O vs. 84.8 ± 23.9 cmH_2_O, *p* < 0.01 and 21.9 ± 14.1 cmH_2_O vs. 73.5 ± 16.5 cmH_2_O, *p* < 0.01 respectively). Greater striated muscle fiber content of controls vs. SUI group (21.6% ± 4.8% vs. 6.1% ± 3.4%, *p* = 0.046). SUI group had higher connective tissue content. SUI group had significantly more fibrillation potentials, fewer motor unit potentials, a higher percentage of polyphasia, and lower EMG amplitude than controls (*p* ≥ 0.01)	Unadjusted. Significantly greater age, menopausal status and BMI in SUI group than controls, parity similar between groups
Henriksson et al. (1979a) [96] Sweden	SUI: *N* = 10. Controls: *N* = 5. Total mean age and range = 54 (38–74) years. SUI confirmed with a positive Bonney test and using urethrocystometry	MUP, MUCP, intravesical pressure, functional urethral length	Urethrocystometry and cinefluorography standing and recumbent with 300 ml of saline solution in bladder	Moderate	Low	No	MUP, MUCP and functional urethral length lower in SUI group than controls. Intravesical pressure similar between groups	Unadjusted. Parity and BMI not reported and age by group not reported
Henriksson et al. (1979b) [97] Sweden	SUI: *N* = 85. Controls: *N* = 42. Total age range = 30–69 years. Mean body mass: 59.5–66.9 kg. SUI confirmed through history and urodynamics	Bladder pressure, MUP, urethral closure pressure, absolute urethral length, functional urethral length	Urethrocystometry and cinefluorography standing and supine with 300 ml of saline solution in bladder	High	Low	No	Similar bladder pressure between SUI group and controls. MUP and urethral closure pressure significantly lower in SUI group than controls (*p* < 0.05) in all age groups except (60–69 year old). Functional and absolute urethral lengths significantly lower in SUI group than controls (*p* < 0.05) in 30–49 year olds, but not in 50–69 year olds. Functional urethral length diminished with age in SUI group but not in controls. No change with age in absolute urethral length in either group	Unadjusted. Both groups subdivided by age. Similar weight and parity between SUI and control groups
Hilton (1983) [98] UK	SUI: *N* = 120 and age = 49.4 (31–84) years. Controls: *N* = 20 and age = 46.1 (25–74) years. SUI confirmed through urodynamics	Functional urethral length, MUP, MUCP, minimum urethral closure pressure	Urodynamics using a microtransducer catheter at rest and during stress (coughing)	High	Low	No	Controls had significantly higher minimum urethral closure pressure, MUP and MUCP than SUI group at rest and during stress (*p* < 0.05). Greater functional urethral length of controls than SUI group during stress (*p* > 0.05) but not at rest	Unadjusted. Mean parity 2.6 (0–10) in SUI group and 1.7 (0–6) in controls. BMI not reported. Slightly older age range in SUI group
Hoffmann and Ulrich (1966) [99] Denmark	SUI: N = 7 and age = 48.4 (33–54) years. Controls: *N* = 14 and age = 40.4 (28–55) years. SUI confirmation not reported	Depression of the base of the bladder during straining, funneling, posterior urethrovesical angle (PUVA, definition not provided)	Cystourethrography	High	Low	No	Average depression of the base of the bladder during straining did not differ between SUI group (15 (5–35) mm) and controls (16 (10–35 mm)). Bladder neck funneling in 1/7 of SUI group and 1/14 in control group. Greater PUVA in controls than SUI group during straining [152° (90–180°) vs. 130° (100–180°)]	Unadjusted. BMI not reported. Parity of SUI group: 2.4 (0–4) and controls: 1.6 (0–4). SUI group older than controls
Howard et al. (2000) [54] USA	SUI (parous): *N* = 23; age = 31.9 ± 3.9 years and BMI = 25.8 ± 5.1 kg/m^2^. Nulliparous controls: N = 17; age = 31.3 ± 5.6 years and BMI = 23.5 ± 2.9 kg/m^2^. Primiparous controls: *N* = 18; age = 30.4 ± 4.3 years and BMI = 24.9 ± 4.3 kg/m^2^. SUI confirmed with a paper towel test and a stress test	Bladder neck position at rest and during displacement with cough and Valsalva; abdominal pressure; stiffness of the vesical neck	Ultrasound, supine, bladder filled to 300 ml. Abdominal pressures recorded simultaneously using an intravaginal microtransducer catheter. Stiffness of vesical neck support calculated by dividing pressure exerted during a particular effort by urethral descent during that effort	Moderate	Low	No	No significant differences in bladder position at rest (*p* = 0.06–0.48). During Valsalva: no significant differences between groups in bladder neck displacement (*p* = 0.42). During cough: greater displacement in SUI group (13.8 ± 5.4 mm) vs. nulliparous controls (8.2 ± 4.1 mm) and primiparous controls (9.9 ± 4.0 mm, *p* = 0.001). Equivalent bladder neck displacement during Valsalva and cough in SUI group (*p* = 0.49), less bladder neck displacement during cough than Valsalva in both control groups (*p* = 0.001–0.002). Greater pressures in cough than Valsalva, but no differences between groups. Stiffness significantly greater in nulliparous controls than primiparous controls and SUI group in cough only (*p* = 0.001)	Unadjusted. Controls age-matched to SUI group ± 5 years. BMI not significantly different between groups. Mean parity of primiparous and SUI group not reported
Hoyte et al. (2004) [100] USA	SUI: N = 10 and age = 48 (38–56) years. Controls: N = 10 and age = 51 (41–69) years. SUI confirmed urodynamically	Maximum thickness, minimum thickness and presence or absence of gaps in the levator ani	Magnetic resonance imaging (MRI) based 3-dimensional color thickness mapping	High	Low	No	Right anterior maximum and minimum thickness of levator ani significantly less in SUI group than control (7.33 mm (5.33–10.01 mm) vs. 10.01 mm (8.6–11.35 mm), *p* = 0.001 and 3.33 mm (0.66–3.33 mm) vs. 4.66 mm (2.00–7.33 mm), *p* = 0.004 respectively). Significantly greater frequency of gaps in the left levator ani in the SUI group than controls (80% vs. 10%, *p* = 0.005)	Unadjusted. Larger age range in SUI group than controls. BMI and parity not reported
Junginger et al. (2018) [41] Germany	SUI: *N* = 68; age = 47 (28–77) years and BMI = 24 (18–50) kg/m^2^. Controls: N = 14; age = 31 (21–52) years and BMI = 23 (18–27) kg/m^2^. SUI confirmed with questionnaire	Intra-abdominal pressure, bladder neck elevation and urethral pressure, EMG amplitude (μV)	Maximal PFM contraction while upright as long as possible, followed by a submaximal contraction, controlled by vaginal EMG (electrodes on sponge). Bladder neck position measured with perineal ultrasound, IAP, urethral pressure with a microtip catheter, and breathing with a circular thorax sensor	High	High	Yes	No significant differences between groups	Unadjusted. Not matched for age, BMI or parity. Controls were significantly younger and had a significantly lower BMI than SUI, parity was similar
Kenton et al. (2011) [66] USA	SUI: N = 37; age = 48 ± 9 years and BMI = 29 ± 7 kg/m^2^. Controls: *N* = 30; age = 39 ± 14 years and BMI = 28 ± 9 kg/m^2^. SUI defined using a questionnaire	Striated urethral sphincter motor unit potential morphology and firing characteristics	Urethral EMG measured using concentric needle electrodes, and urodynamic testing done using a Laborie Dorado urodynamic equipment and microtip catheters	High	Low	No	SUI group had lower amplitude motor unit potentials, fewer turns per second in the EMG interference pattern, lower turns/amplitude ratios in motor unit potentials recorded from the striated urethral sphincter. They also had lower percent activity (defined as percent of time during an epoch that sharp activity occurs), and fewer short segments (portion of EMG signal with sharp activity characterized by short rise times) than controls. In a multivariable model, activity of the striated urethral sphincter during bladder filling was significantly lower in SUI group than in controls (*p* = 0.001)	Adjusted for age, race, continence status, and vaginal parity. Controls were, on average, a decade younger and were more likely to be vaginally parous than SUI group. BMI similar between groups
Kim et al. (2003) [101] South Korea	SUI: *N* = 63 and age = 54 ± 9 years. Controls: *N* = 16 and age = 40 ± 12 years. SUI determined through history, physical examination findings, and urodynamic evaluation	Thickness of the striated muscle, smooth muscle, and mucosa–submucosa of the urethra; degree of asymmetry of the puborectalis muscle; frequency of distortion in the periurethral, paraurethral, and pubourethral ligaments; degree of the vesicourethral angle; and dimension of the retropubic space	Magnetic resonance imaging with an endovaginal coil	High	Low	No	Striated muscle layer of the urethra significantly thinner in SUI group than in controls (1.9 ± 0.5 mm vs. 2.6 ± 0.4 mm, *p* < 0.001). No significant difference in smooth muscle or mucosa-submucosa thickness between groups. A high degree of asymmetry of puborectalis muscle (> 1.5) was more frequent in SUI group (29%) than in controls (0%, *p* = 0.015). Greater frequency of distortion of all ligaments in SUI group vs. controls (*p* < 0.01). Greater vesicourethral angle in SUI group than controls (148° ± 13° vs. 122° ± 11°, *p* < 0.001). Greater retropubic space in SUI group than controls (7.5 ± 1.6 mm vs. 5.1 ± 1.1 mm, *p* < 0.001)	Unadjusted. SUI group significantly older than controls. Not matched for body weight (not reported) and parity not reported
Kirschner-Hermanns et al. (1993) [23] Germany	SUI: *N* = 24. Controls: *N* = 6. Total age range = 25–55 years. Method of determining SUI not reported	Levator ani muscle composition and morphology	Magnetic resonance imaging (MRI)	High	Low	No	Degeneration of levator ani muscle in ≥ 45% of SUI group, but not controls. PFM tissue partially replaced with fatty and connective tissue. Sharp dorsal angulation of levator ani lost in 16/24 of SUI group and 0/6 controls	Unadjusted. BMI and parity not reported. Age range for individual groups not reported. In abstract 45% of SUI group had degeneration of levator ani but reported as 66% in results section
Kirschner-Hermanns et al. (1994) [102] Germany	SUI: *N* = 32. Controls: *N* = 12. SUI determined through history, physical examination, radiographic and urodynamics	Area of urethral sphincter muscle and sphincter circumference	B-mode Intra-urethral ultrasound. Position and bladder filling not reported	High	Low	No	Area of urethral sphincter muscle smaller in SUI group than control (0.73 ± 0.4 cm^2^ vs. 1.03 ± 0.3 cm^2^, *p* < 0.05). Smaller sphincter circumference in SUI group than controls 2.97 ± 0.71 cm^2^ vs. 3.61 ± 0.5 cm^2^, *p* < 0.05)	Unadjusted. BMI, parity and age not reported
Koenig et al. (2017) [29] Switzerland	SUI: *N* = 50; age = 41.7 ± 10.9 years and body mass = 63.2 ± 12.8 kg. Controls: N = 20; age = 30.0 ± 4.7 years and body mass = 30.0 ± 4.7 kg. SUI confirmed with self-report	EMG amplitude (μV) at rest and during MVCs, ICC, SEM and minimal detectable difference	Surface EMG with Periform™ probe. Two MVCs for 5 s with 1- min rest in between. Onsets determined using a threshold method and visually to establish 5 s window of contraction.	High	High	Yes	Raw EMG amplitude (μV) higher in controls than and SUI group, but large variability and large minimal detectable differences (21–44%). SUI group often unable to hold a PFM contraction for 5 s making onset determinations difficult. High ICCs; 0.78–0.99, high SEM: 7.5–15.7%	Unadjusted. SUI group significantly older than controls. Body mass not significantly different between groups. Parity not reported
Kondo et al. (1994) [103] Japan	SUI: *N* = 26 (13/26 age matched); age = 51 (32–71) years and mass 56.9 ± 9.0 kg. Controls: *N* = 21; age = 44 (33–51) years and mass = 52.6 ± 7.1 kg. SUI determined through urodynamics	Vaginal wall strength and rectus fascia shear strength (kg) left and right side	Digital force gauge used intra-operatively to determine shear force through repeated application to the vaginal wall and rectus fascia bilaterally	High	Low	No	Irrespective of age and laterality shear strength of anterior vaginal wall lower in SUI group than controls (*p* < 0.01). Irrespective of age shear strength of rectus fascia lower in SUI group than in controls on the left (*p* < 0.05) and right (*p* < 0.01)	Subgroup of SUI group (n = 13) matched for age with control group. SUI group had higher body mass than control group, but not significant. Parity did not differ between groups
Kondo et al. (2001) [104] Japan	SUI: *N* = 55 and age = 61.4 ± 1.4 years. Controls: *N* = 19 and age = 62.3 ± 2.9 years. SUI confirmed with questionnaire, physical examination, cystometry, 1-h pad test	Mid urethra thickness at the peripheral zone and central portion	Transvaginal B-mode ultrasound, supine. Bladder filling not reported	High	Low	Yes	At the peripheral zone of the urethra the mean mid- urethra thickness less in SUI group (2.14 ± 0.04 mm) compared to controls (2.78 ± 0.08 mm, reported as a significance difference in abstract, but not in results). No difference in thickness in central zone between groups	Unadjusted. No significant difference in age between groups. BMI and parity not reported
Kuhn et al. (2008) [105] Switzerland	SUI: *N* = 189; age = 56 (28–95) years and BMI = 29 (22–38) kg/m^2^. Controls: *N* = 15; age = 47 (23–62) years and BMI = 27 (24–32) kg/m^2^. SUI confirmed with clinical and urodynamics	Urethral retro resistance pressure (the retrograde infusion of sterile fluid against a closed sphincter), maximum urethral closure pressure	Urodynamics using a microtip transducer catheter	High	Low	No	Maximum urethral closure pressure and urethral retro resistance pressure were significantly higher in healthy individuals than in women with SUI (*p* = 0.0001)	Unadjusted. SUI group were older and had higher BMI than controls. Parity did not differ between groups
Kuo (1998) [106] Taiwan	SUI: *N* = 60 and 54.2 ± 11.9 years for women with mild SUI and 57.7 ± 11.4 years for women with severe SUI Controls: N = 14 and age = 53.3 ± 17.6 years. SUI determined from self-report	Cross-sectional area of the urethral and paraurethral structures	Transrectal sonography of the urethra, standing during rest and straining with a full bladder and uroflowmetry	High	Low	No	CSA of mid-urethra similar between SUI group and controls (86.7 ± 29.9 mm^2^ vs. 96.4 ± 38.4 mm^2^, *p* > 0.05). No significant differences in smooth or striated muscle components of the midurethral CSA between groups, or in thickness of pubourethral ligament. Thickness of urethropelvic ligaments significantly thinner in SUI group than in controls (5.9 ± 1.7 mm vs. 10.1 ± 2.7 mm, *p* < 0.001). In 35.7% of controls, in 16.7% of mild SUI and in 5.3% of severe SUI, complete surrounding of the urethra was seen. Bladder neck incompetence was seen in 42 of SUI group but in no controls	Unadjusted. No significant differences in age or parity. BMI not reported. Several additional comparisons with respect to SUI include a combination of controls and individuals with frequency-urgency syndrome
Leitner et al. (2016) [36] Switzerland	SUI: *N* = 22; age = 45.3 ± 9.5 years and BMI = 21.4 ± 2.0 kg/m^2^. Controls: *N* = 28; age = 38.7 ± 10.0 years and BMI = 21.8 ± 1.7 kg/m^2^. SUI determined using ICIQ-UI SF and self-report	EMG amplitude (%MVC), time of maximum EMG amplitude and onset time. PFM strength (Modified Oxford Scale)	Surface EMG recorded with the STIMPON™ vaginal probe during running on a treadmill at speeds of 7, 11, and 15 km/h. PFM strength assessed manually	High	Moderate	No	No statistically significant differences in EMG activity of the PFMs between continent and incontinent women, regardless of running speed or phase of the gait cycle. No difference in PFM strength between women with and without SUI	Unadjusted. Incontinent women were on average 6.5 years older than controls. No difference in BMI between groups. Parity not recorded
Leitner et al. (2017) [107] Switzerland	SUI: N = 19; age = 45.3 ± 10.3 years and BMI = 21.6 ± 2.0 kg/m^2^. Controls: *N* = 27; age = 38.7 ± 10.4 years and BMI = 21.7 ± 1.7 kg/m^2^. SUI was confirmed with ICIQ-UI SF	Translational (cranial-caudal) and rotational (backward- forward about the lateral axis) displacement of the vaginal probe	Three-dimensional pelvic floor kinematics during running assessed with electromagnetic tracking system attached to STIMPON™ vaginal probe	High	Moderate	No	Translation and rotation of the probe did not differ between SUI groups and controls (*p* > 0.05)	SUI group significantly older than controls, BMI not significantly different between groups. Parity not recorded
Leitner et al. (2019) [37] Switzerland	SUI: *N* = 22; age = 45.9 ± 9.7 years and BMI = 21.5 ± 2.0 kg/m^2^. Controls: *N* = 28; age = 38.9 ± 10.3 years and BMI = 21.7 ± 1.8 kg/m^2^. SUI was confirmed with ICIQ-UI SF	Linear regression calculated for rate of activity from onset to peak, peak to offset, and within 200 ms after both onset and peak. Peak EMG activity and timing variables related to onset, peak and offset	PFM EMG recorded with STIMPON™ vaginal probe during rest, maximum voluntary contractions (MVC), and five FVC. MVC peak activity was used to normalize EMG data	High	Moderate	No	Significantly longer relaxation times in SUI group vs. controls in fast voluntary contraction. Onset to offset: 1586.4 ± 557.0 ms for SUI and 1247.5 ± 444.5 ms for controls (*p* = 0.02). Peak +200 ms: −74.4 ± 53.9 %MVC/s for SUI and − 120.7 ± 83.5 %MVC/s for controls (*p* = 003)	Unadjusted. SUI group significantly older than controls, BMI not significantly different between groups. Parity not reported
Lemos et al. (2018) [108] Brazil	SUI: N = 20; age = 47.1 ± 7.8 years and BMI = 26.7 ± 4.4 kg/m^2^. Controls: N = 20; age = 43.5 ± 8.4 years and BMI = 24.9 ± 2.5 kg/m^2^. SUI verified by means of Pad Test above 1 g	PFM function: Power (Oxford scale), endurance, number of repetitions at same force and number of fast repetitions. MVC, mean of basal EMG amplitude (μV) for 1 min, peak of the greater of 10 phasic contractions and best of five tonic contractions sustained for 5 s. Anterterior pelvic tilt angle	Palpation from a therapist. Measurements in lying and standing. Surface EMG electrodes attached in perianal region and two over right internal oblique muscle. Pelvic tilt from styrofoam balls on ASIS and PSIS digitized from still photographs	High	High	Partially	Significantly less power lying down on OXFORD scale in SUI group (median (IQR)): 3.0 (3.0–40) vs. controls (4.0 (3.0–4.0), *p* = 004). Significantly less EMG amplitude in several variables in SUI group vs. control (*p* = 0.01). Greater anterior pelvic tilt in SUI group mean ± SD vs. controls −17° ± 4° vs. −14° ± 5° (*p* = 0.01), respectively. Moderate significant correlations with some EMG variables (r = 046–0.51, *p* = 0.02–0.04) in SUI group but not controls	Unadjusted. No significant differences in age, BMI or parity
Li et al. (2017) [109] China	SUI: *N* = 87; age = 56.5 ± 10.6 years and BMI = 25.1 ± 3.3 kg/m^2^. Controls: *N* = 72; age = 55.1 ± 8.0 years and BMI = 23.6 ± 2.7 kg/m^2^. SUI group were clinically diagnosed and scheduled for surgery	Detrusor wall thickness, bladder neck descent, proximal urethral rotation angle (α angle) posterior vesicourethral angle (β angle), and the formation of a funnel-shaped urethra	Transperineal two-dimensional ultrasound, supine with an empty bladder (< 50 ml) at rest? And? During Valsalva maneuver	High	Low	No	No difference in detrusor wall thickness between groups. Bladder neck descent greater in SUI group than control group (21.9 ± 8.0 mm vs. 11.4 ± 6.6 mm, *p* < 0.001). α and β angles significantly larger in SUI group (50.14° ± 23.68° and 162.75° ± 17.17°, respectively, *p* < 0.001) than in controls (28.49° ± 14.45° and 122.28° ± 13.90°, respectively, *p* < 0.001). Prevalence of a funnel-shaped urethra significantly higher in SUI group (94%) than in controls (3%)	Matched for age. The control group had lower BMI and lower parity than SUI group
Li et al. (2018) [110] China	SUI: N = 15; age = 28.5 ± 2.9 years and BMI = 21.9 ± 1.7 kg/m^2^. Controls: *N* = 35 (parous, additional 35 nulliparous); age = 26.7 ± 2.2 years and BMI = 22.3 ± 3.0 kg/m^2^. SUI confirmed with self-report and physical examination	Levator ani muscle (LAM) injury, vesical neck movement, urethral length and mobility and urethral sphincter dysfunction (bladder neck funneling)	MRI at rest and Valsalva maneuver. LAM injury evaluated using scoring system by DeLancey et al. (2003). Locations of vesical neck at rest and Valsalva measured as vertical distance to pubococcygeal line and movement defined as difference between rest and Valsalva status	Moderate	Low	No	Significantly greater percentage of LAM injury in SUI group (60%) vs. controls (51.4%, *p* = 0.009). During Valsalva: significantly greater magnitude of vesical neck location in SUI group (−9.6 ± 5.2 mm) vs. controls (−4.2 ± 11.3 mm, *p* = 0.002) and significantly greater vesical neck movement (28.5 ± 6.3 mm vs. 24.2 ± 11.5 mm, *p* = 0.006, for SUI and controls respectively. Significantly greater percentage of bladder neck funneling in SUI group (80%) vs. controls (22.9%, *p* < 0.001)	Unadjusted. BMI significantly lower in SUI group than parous controls. No difference in age between groups. Mean parity not reported Bladder neck funneling in parous controls reported as 22.9% in table but 21.2% in text
Li et al. (2020) [111] China	SUI: *N* = 33; age = 60.6 ± 10.3 years and BMI = 24.6 ± 1.6 kg/m^2^. Controls: *N* = 25 (perimenopausal, additional 25 nulliparous); age = 45.9 ± 6.6 years and BMI = 24.1 ± 3.1 kg/m^2^. SUI confirmed with positive stress induction test, positive bladder neck elevation test, and positive cotton swab test	Periurethral ligament, pubovisceral and puborectalis muscle defects graded on a 3-point scale from normal-disruption. (1) Urethral length, (2) functional urethral length, (3) urethral hypermobility (UH), (4) bladder funneling, (5) urethra opening, (6) POP, (7) H line (length of levator hiatus) and M line (descent of pelvic floor). Cystocele (bladder descent)	Static and dynamic MRI (cycle of rest, squeezing, straining and defecation).	High	Low	No	Greater percentage of periurethral ligament defects in SUI (76%) vs. perimenopausal controls (32%, *p* < 0.001). Greater percentage of pubococcygeal muscle defects in SUI (66%) vs. perimenopausal controls (28%). Shorter urethra length in SUI vs. perimenopausal controls (31.8 ± 5.0 mm vs. 34.4 ± 3.5, respectively, *p* = 0.03). Shorter functional urethra length at rest in SUI vs. perimenopausal controls (18.0 ± 4.4 mm vs. 23.4 ± 3.9 mm, *p* < 0.001) and during defecation (2.2 ± 5.2 mm, vs. 13.0 ± 5.9 mm, *p* < 0.001). Greater proportion of bladder neck funneling during defecation in SUI (27/33) vs. perimenopausal controls (3/25, *p* < 0.001). Greater H line, M line and presence of cystocele in SUI vs. perimenopausal controls during defecation (*p* < 0.001–*p* = 0.01)	Unadjusted. SUI group older than controls. Similar parity and BMI between groups
Liang et al. (2006) [112] Taiwan	SUI: N = 29 and age = 51.7 ± 10.7 years. Controls: N = 28 and age = 48.0 ± 14.1 years. SUI defined through clinical and urodynamic testing	Periurethral vascularization and blood flow (vascularization index, flow index and vascularization-flow index)	Three-dimensional power Doppler ultrasonography, supine. Bladder filling not reported	Moderate	Low	No	Fewer periurethral vessels and lower flow in SUI group than controls based on a lower vascularization index (median: 0.60 vs. 0.35, *p* = 0.003), lower flow index (median: 21.20 vs. 22.86, *p* = 0.027) and lower vascularization-flow index (median: 0.072 vs. 0.147, *p* = 0.005)	Unadjusted. Similar age and parity of groups. BMI not reported
Lukanovič and Patrelli (2011) [113] Slovenia	SUI: *N* = 100 and age = 46.2 ± 8.5 years. Controls: N = 50 and age = 53.8 ± 10.9 years. SUI confirmed through clinical and urodynamic evaluation	Urethrovesical junction position at rest and mobility during coughing, the distance between the urethrovesical junction and inferior edge of the symphysis pubis and the distance between the urethrovesical junction and the vertical plane of the front edge of symphysis pubis	Urodynamic measurement and 2D transperineal B-mode ultrasound, supine position, bladder filled with 300 ml saline solution	High	Low	No	No difference in distance of the bladder neck to symphysis pubis at rest in the horizontal plane or the displacement of the bladder neck in the horizontal plane during coughing between groups. In the vertical plane mean distance of the bladder neck at rest significantly more cranial in SUI group (16.1 ± 4 mm) compared to controls (7.9 ± 2.9 mm, *p* = 0.001)	Unadjusted. Women with SUI were younger, had higher parity and were more commonly menopausal than controls. BMI not reported
Macura et al. (2015) [114] USA	SUI: *N* = 21; age = 54.4 ± 11.8 years and BMI = 29.95 ± 6.6 kg/m^2^. Controls: *N* = 10; age = 45.1 ± 13.6 years and BMI = 29.98 ± 7.0 kg/m^2^. SUI confirmed urodynamically	Urethral angle (angle between the patient body axis and the axis of the urethra); bladder neck descent during strain; morphology of the periurethral ligaments; vaginal shape; urethral sphincter integrity, urethral length, urethral muscle thickness at mid-urethra; presence and extent of bladder neck funneling; morphology of the puborectalis muscle; and pubovaginal distance	High-resolution endocavitary MRI and dynamic pelvic floor MRI	High	Low	Yes	Pubovaginal distance and periurethral ligament disruption were significantly associated with incontinence. Periurethral ligament symmetry reduces the odds of incontinence by 87%. Bladder neck funneling and suprapubic urethral sphincter length were significantly associated with SUI. The presence of bladder neck funneling reduced the odds of urethral hypermobility by almost 95%. Higher suprapubic urethral sphincter length at rest was associated with urethral hypermobility. In multivariable model, only periurethral ligament disruption was significantly more common in incontinent women than in controls	Adjusted for MRI variables. Age, ethnicity, BMI and parity did not significantly differ between groups. A history of obstetrical trauma (episiotomy, forceps delivery, perineal laceration) was more common in women with SUI than in controls (52% vs. 0%)
Madill et al. (2009) [34] Canada	SUI: *N* = 44 (33 mild and 11 moderate to severe); mild SUI age = 46.5 ± 7.4 years, BMI = 27.0 ± 5.3 kg/m^2^; moderate to severe SUI age = 52.8 ± 8.4 years and BMI = 28.4 ± 5.7 kg/m^2^. Controls: *N* = 28 age = 46.8 ± 7.6 years and BMI = 24.3 ± 3.4 kg/m^2^. SUI (severity) confirmed with 3-day bladder diaries	Maximum EMG RMS amplitude (μV), lower and upper vaginal pressure, time lag between abdominal muscles and PFM, average pressure versus EMG curves	Surface EMG of PFM recorded with Femiscan™ probe, internal and external oblique and rectus abdominus muscles recorded using adhesive electrodes and intravaginal pressure recorded using custom air-filled sensors during maximal PFM contractions	Moderate	High	Yes	Significantly higher PFM and external and internal oblique EMG amplitudes in controls than SUI groups (*p* < 0.05). Delayed activation of rectus abdominus in moderate-severe SUI group. No difference between groups in intravaginal pressure and similar intravaginal pressure versus EMG curves between groups	Unadjusted. Significantly larger BMI in SUI group and more parous women in SUI group compared to controls. Moderate-severe group older than other groups
Madill et al. (2010) [33] Canada	Mild SUI: *N* = 8; age = 52.3 ± 7.0 years and BMI = 27.1 ± 5.1 kg/m^2^. Moderate to severe SUI: N = 8; age = 53.5 ± 6.0 years and BMI = 27.4 ± 5.4 kg/m^2^. Controls: N = 8; age = 51.6 ± 6.2 years and BMI = 25.0 ± 3.7 kg/m^2^. SUI confirmed through questionnaire and detrusor overactivity ruled out by urodynamic testing	Peak EMG amplitude minus baseline amplitude (μV), peak posterior vaginal wall (PVW) pressure minus baseline pressure, rate of pressure generation, timing of peak intra-abdominal pressure and EMG relative to peak PVW pressure, slope of PVW pressure vs. EMG curve and intercept of PVW vs. EMG curve	Three maximum coughs in supine and standing. PFM EMG recorded with Femiscan™ probe; abdominal EMG recorded using adhesive electrodes. Pressure was recorded via two air-filled pressure transducers, one placed adjacent to the anterior vaginal wall and the other placed adjacent to the posterior vaginal wall	High	High	Yes	Maximum PFM EMG amplitude and PVW pressures did not differ between groups. External oblique (EO) and internal oblique (IO) EMG amplitudes: continent women produced higher EO EMG amplitudes (*p* < 0.001 for both), while women with mild SUI produced higher IO EMG amplitudes (*p* = 0.008 vs. continent, *p* < 0.001 vs. moderate to severe SUI).Women with moderate- severe SUI generated peak PVW pressure at more slowly than controls and the mild SUI group (*p* = 0.025 and *p* = 0.022, respectively)	Matched for age, BMI and parity
Mattiasson and Teleman (2006) [115] Sweden	SUI: *N* = 50. Controls: *N* = 28. Age range: 53–63 years. SUI determined through clinical examination	Maximum urethral pressure, maximum urethral closure pressure, observation of a pressure fall during or immediately following squeeze, acceleration of urinary flow and maximal urinary flow rate	Pressure and flow measurements made using a microtip transducer catheter	Moderate	Low	Yes	Lower MUP and MUCP in SUI group compared to controls (61.0 ± 21.4 cmH_2_O vs. 67 ± 20.0 cmH_2_O and 46.3 ± 18.9 cmH_2_O vs. 52 ± 18.0 cmH_2_O, respectively, direct statistical comparisons not made). SUI group more often had a pressure fall during or immediately following squeeze than controls (65.2% vs. 25% of groups). Acceleration of urinary flow lower in SUI group than controls (12.5° ± 13.8° vs. 32° ± 24.9°). Maximal flow rate higher in SUI group than controls (29.8 ± 34.6 ml/s vs. 16 ± 8.2 ml/s)	Unadjusted. SUI group compared to controls of same age and parity. BMI not reported
Meyer et al. (1996a) [116] Switzerland	SUI: *N* = 279 SUI (38 with low urethral pressure and 241 with normal urethral pressure) and age = 57 ± 9 years 49 ± 12 years. Controls: N = 7 and age = 38 ± 10 years or 39 ± 11 years, discrepancy between table and text. SUI determined through patient history (Ingelmann-Sundberg classification)	Functional urethral length, MUCP, urethral sphincter motor unit potential duration and amplitude, pudendal motor latencies to the urethral sphincter, area of response to stimulation and incidence of polyphasic potentials	Needle EMG, functional urethral length and MUCP recorded using a microtip transducer and urodynamics with a full bladder during supine and standing	High	Low	No	Functional urethral length did not differ between groups. MUCP closure pressure lower in SUI group than in controls in both supine and standing, particularly in SUI with low urethral pressure (18 ± 9 cmH_2_0 vs. 90 ± 43 cmH_2_0 in standing, *p* = 0.0004). In SUI group with low urethral pressure with respect to controls: longer urethral sphincter motor unit potential duration and pudendal motor latencies (*p* ≤ 0.05); smaller area of response to stimulation (*p* = 0.009); greater incidence of polyphasic potentials (*p* = 0.02); no difference in motor unit amplitudes (*p* = 0.4)	Unadjusted. Parity similar. BMI not reported. SUI group older than controls
Meyer et al. (1996b) [117] Switzerland	SUI: N = 32 and age = 57 ± 9 years 48 ± 10 years. Controls: *N* = 74 and age = 34 ± 12 years. Confirmation of SUI not described, had not yet been investigated urodynamically	Bladder neck position and displacement: Distance from bladder neck and central line of pubic symphysis and distance from bladder neck and axis constructed perpendicular to central line of pubic symphysis	Transperineal ultrasound supine and standing at rest and under stress (Valsalva), bladder filled to more than 200 ml	High	Low	No	Bladder neck position lower in SUI group than controls in supine and standing (*p* < 0.0005). Greater bladder neck displacement between rest and Valsalva in supine and standing in SUI group than controls (*p* < 0.05), e.g., change in distance from bladder neck and central line of pubic symphysis 11 ± 5 mm and 7 ± 5 mm in SUI group and controls, respectively (*p* < 0.0005)	Unadjusted. BMI not reported. SUI group older. Controls were nulliparous and SUI group had mean parity of 2.4
Moser et al. (2018) [38] Switzerland	SUI: *N* = 22; 45.3 ± 9.5 years and BMI = 21.4 ± 2.0 kg/m^2^. Controls: *N* = 28 age = 38.7 ± 10.0 years and BMI = 21.8 ± 1.7 kg/m^2^. SUI confirmed with ICIQ-UI SF and personal history	EMG amplitude (%MVC) and timing before and after jumps	Surface EMG of PFMs recorded with STIMPON™ probe during drop jumps and counter movement jumps	High	Moderate	No	No significant differences between groups	SUI group significantly older than controls, BMI not significantly different between groups. Parity not recorded
Moser et al. (2019) [118] Switzerland	SUI: N = 22; 45.8 ± 9.9 years and BMI = 21.4 ± 2.0 kg/m^2^. Controls: N = 28 age = 39.3 ± 10.5 years and BMI = 21.5 ± 1.7 kg/m^2^. SUI confirmed with ICIQ-UI SF and personal history	PFM displacement (craniocaudal translation and forward-backward rotation)	Electromagnetic tracking system assessed PFM displacement with 6 degrees of freedom using STIMPON™ vaginal probe	High	Moderate	No	No significant differences between groups	SUI group significantly older than controls, BMI not significantly different between groups. Parity not recorded
Najjari et al. (2016) [119] Germany	SUI: N = 72 and age = 62 ± 11 years. Controls: *N* = 32 and age = 62 ± 12 years. No details provided in terms of how SUI was diagnosed	Total urethral length and functional urethral length	Ultrasound supine at rest, during contraction and Valsalva maneuver. Urodynamic assessment performed using a triple lumen catheter with a pressure sensor (transducer). Bladder filled (water) to approximately 300 ml	Moderate	Low	No	Total urethral length longer in SUI group (38.5 ± 6.8 mm) than the control group (28.7 ± 3.8 mm, *p* < 0.01). Functional urethral length shorter in SUI group (22.4 ± 7.5 mm) than control group (24.2 ± 6.5 mm, *p* < 0.01)	Unadjusted. BMI and parity not reported. Average age similar between groups
Olgan et al. (2016) [120] Turkey	SUI: *N* = 37; age = 57.4 ± 7.7 years and BMI = 29.3 ± 4.3 kg/m^2^. Controls: *N* = 77; age = 57.0 ± 7.0 years and BMI = 28.9 ± 3.5 kg/m^2^. SUI determined using a questionnaire	Echogenicity of the mid-urethra using grayscale histograms.	2D transvaginal B-mode ultrasound. Position and bladder filling unclear	Moderate	Low	Yes	Tissue echogenicity (grayscale) lower in posterior mid-urethra in SUI group (53.46 ± 16.20) than controls (62.92 ± 18.07, *p* = 0.008) and higher in anterior mid-urethra region in SUI group (79.69 ± 16.16) than in controls (57.35 ± 15.11, *p* = 0.001)	Adjusted for parity, history of operative delivery, macrosomic baby and smoking. Age, menopause duration, weight and BMI did not differ between groups. SUI group had higher parity than controls
Oliveira et al. (2007) [121] Brazil	SUI: *N* = 21 (multiparous). Controls: *N* = 42 (21 nulliparous and 21 multiparous) Total age: 34.4 (18–50) years. SUI confirmed urodynamically	Cross-sectional area of the levator ani muscles, Doppler velocimetric parameters of the vessels within the levator ani muscle and frequency of absent end diastolic shift	Transperineal B-mode and Doppler ultrasound, supine. Bladder filling not reported	High	Low	No	Cross-sectional area of the levator ani muscles was higher in parous controls than in SUI group (1.78 cm^2^ vs. 1.32 cm^2,^ *p* < 0.05). No differences in Doppler velocimetric parameters between groups. Frequency of absent end diastolic shift was higher in SUI group than in controls (22.2% vs. 7.1%, *p* < 0.05)	Unadjusted. Mean age and BMI not reported for each group, however age and BMI said to be homogenous between the SUI group and multiparous controls
Peng et al. (2007) [60] USA	SUI: *N* = 10; age = 51.5 ± 5.3 years and BMI = 24.98 ± 4.11 kg/m^2^. Controls: *N* = 23; age = 39.0 ± 2.3 years and BMI = 22.4 ± 1.99 kg/m^2^. SUI defined by self-report and questionnaires	Maximum vaginal pressures measured at the anterior, posterior and lateral vaginal walls, relative difference in pressure between locations and position of maximum vaginal pressure	Pressures measured during maximum effort voluntary PFM contraction using a custom pressure sensor inserted intravaginally	High	Low	Yes	Maximum posterior vaginal pressure of the controls was significantly greater than that of the SUI patients both at rest (3.4 ± 0.3 N cm^−2^ vs. 2.01 ± 0.36 N cm^−2^, *p* < 0.05) and during PFM contraction (4.18 ± 0.26 N·cm^−2^ vs. 2.25 ± 0.41 N·cm^−2^, *p* < 0.01). Difference between the posterior and anterior vaginal walls was significantly in controls (*p* < 0.05), but not in the SUI group. No difference in the position of maximum pressure between groups (*p* = 0.16)	Unadjusted. SUI group older than controls. Similar parity and no significant difference in BMI between groups
Pizzoferrato et al. (2017) [122] France	SUI: *N* = 358; age = 40.2 ± 12.1 years to 63.3 ± 15.4 years (mild-severe) and BMI = 21.8 ± 2.8 kg/m^2^ to 28.1 ± 6.8 kg/m^2^ (mild-severe). Controls: N = 42; age = 45.1 ± 14.9 and BMI = 24.1 ± 3.9 kg/m^2^. SUI determined using ICIQ-UI SF and the Mesure du Handicap Urinaire (MHU)	Bladder neck mobility, strength of levator ani (0–5), urethral closure pressure, maximum urethral closure pressure	POP-Q, Urodynamic assessment and manual digital palpation	Moderate	Low	No	Bladder neck mobility associated with SUI (odds ratio = 3.32, 95% CI 1.85–5.96) and had good discriminative ability for SUI (AUC = 0.84). Mean urethral closure pressure at stress was 52.3 ± 29.9 cm H_2_O in continent women and 10.5 ± 20.9 cm H_2_O in incontinent women. Urethral closure pressure at stress was associated with SUI (odds ratio = 0.92, 95% CI 0.88–0.98) and had excellent discriminative ability for SUI (AUC = 0.90). Maximum urethral closure pressure at rest had low discriminative ability for SUI (AUC = 0.79). Levator ani muscle strength significantly associated with severity of stress incontinence (odds ratio = 0.78, 95% CI 0.66–0.93)	Odds ratios for bladder neck mobility, urethral closure pressure at stress, and Levator ani muscle strength were controlled for age and BMI. Less parity in controls than SUI group
Pontbriand-Drolet et al. (2016) [19] Canada	SUI: *N* = 22; age = 68.3 ± 5.7 years and BMI = 25.44 ± 2.72 kg/m^2^. Controls: N = 22; age = 66.5 ± 5.0 years and BMI = 24.46 ± 3.88 kg/m^2^. SUI determined with self-report	Bladder neck and urethral sphincter morphology: Pubococcygeal (PC) line, anorectal angle, H-line (from the inferior edge of the pubic symphysis to the apex of the anorectal angle), M-line (perpendicular from the PC line to apex of the anorectal angle), PC/H-line angle, urethrovesical (UV) junction height, uterocervical junction height, UV junction approximation- height, the occurrence of bladder neck funneling and posterior urethrovesical angle	Magnetic resonance imaging (MRI) at rest, PFM MVC, and straining	High	Low	Yes	SUI group had a greater occurrence of bladder neck funneling at rest (81%) than controls (46%, *p* = 0.03). SUI group had larger posterior urethrovesical angle (169.81° ± 42.35°) than controls (138.67° ± 39.58°, *p* = 0.008) at rest. No other significant differences between SUI group and controls	Unadjusted. No statistically significant differences in age, BMI, number of vaginal deliveries or history of hysterectomy between groups
Ptaszkowski et al. (2015) [12] Poland	SUI: *N* = 16; age = 63.9 ± 5.9 years and BMI = 26.7 ± 3.7 kg/m^2^. Controls: *N* = 14; age = 66.1 ± 6.3 years and BMI = 24.9 ± 3.5 kg/m^2^. SUI determined using ICIQ-UI SF	EMG amplitude (μV)	Surface EMG of PFMs, gluteus maximus, adductor muscles and rectus abdominus recorded using a Life-care Vaginal Probe PR-02 in a standing position during resting and functional tasks	High	High	No	No difference in rectified mean amplitude of the EMG signals recorded from the PFMs between groups. Amplitude of EMG activity recorded from rectus abdominis muscle during resting and functional activity significantly higher in SUI group than in controls (*p* < 0.005)	Unadjusted. Groups matched for age, weight, height, and BMI. Parity not reported
Rinne et al. (2010) [123] Finland	SUI: N = 40; age = 51 ± 8.5 years and BMI = 26.4 ± 5.4 kg/m^2^. Controls: *N* = 15; age = 41 ± 5.8 years and BMI = 24.1 ± 3.2 kg/m^2^. SUI confirmed with a cough test	Urethral length, mid-point of the measured total length of the urethra, and bladder neck funneling	Dynamic MRI at rest, during PFM contraction, during coughing and while voiding with a bladder volume of 200 ml	High	Low	No	No significant differences in urethral length at rest or bladder neck funneling between groups. Position of the mid-point of the urethra did not differ between groups at rest but was significantly higher upon the movement caused by straining (*p* = 0.002) and coughing (*p* = 0.01) in controls than in SUI group. On contracting the PFMs the point of the mid-urethra rose higher in the controls than SUI group (*p* = 0.002) and during straining moved more toward the axis of symphysis pubis in the SUI group than in the controls (*p* = 0.002)	Unadjusted. SUI group significantly older than controls. No significant difference in BMI or parity
Sapsford et al. (2008) [27] Australia	SUI: N = 8; age = 41.8 (33–55) years and BMI = 22.8 (19.02–26.20) kg/m^2^. Controls: *N* = 9; age = 45 (32–66) years and BMI = 23.44 (19.55–26.32) kg/m^2^. SUI determined with self-report	EMG amplitude (μV)	Surface EMG of PFM and abdominal muscles using (Periform™ probe) in 3 sitting postures: slump supported, upright unsupported, and very tall unsupported	High	High	No	Non-normalized EMG of the PFMs was lower in women with SUI compared to controls when in an upright unsupported sitting posture (*p* < 0.05). Women with SUI had higher activity in the rectus abdominis muscles than controls when sitting upright without support (*p* = 0.059)	Unadjusted. Similar age, BMI and parity of groups
Schaer et al. (1999) [124] USA	SUI: N = 30 and age = 50 ± 14 years. Controls: N = 28 and age = 36 ± 9 years. SUI confirmation urodynamically	Depth and width of proximal urethral dilation during coughing and Valsalva maneuver, bladder neck dilation and descent, abdominal pressure increases	Transperineal ultrasound, supine, bladder volume 300 ml	High	Low	No	No significant differences in abdominal pressure increases between groups. During Valsalva all SUI group and 12/28 controls had urethral dilation. During coughing, dilation visible in 29/30 SUI group and 6/28 controls Bladder neck descent was seen in both groups	Unadjusted. BMI not reported. In controls nulliparous women did not present with dilation during Valsalva or coughing. SUI group older than controls
Schick et al. (2004) [10] Canada	SUI: *N* = 286. Controls: *N* = 83. Mean combined age = 49.3 ± 13.2 years. SUI determined with self-report (reported that their SUI bothered them enough to seek medical advice)	Maximum urethral closure pressure, urethral hypermobility, urethral incompetence	Multichannel urodynamics. Urethral hypermobility was measured during maximal abdominal straining with an empty bladder	High	Low	No	Maximum urethral closure pressure significantly lower in the incontinent group. Incontinent patients had a greater probability of a higher grade of urethral incompetence and hypermobility. The best single predictor of clinically significant SUI was urethral incompetence, followed by urethral hypermobility and maximum urethral closure pressure	Adjusted for age, maximum urethral closure pressure, urethral hypermobility and urethral incompetence. BMI and parity not reported
Sendag et al. (2003) [125] Turkey	SUI: N = 30; age = 50.5 ± 9.4 years and mass = 69.8 ± 7.4 kg. Controls: *N* = 17; age = 45.4 ± 8.9 years and mass = 65.0 ± 4.6 kg. SUI confirmed with urodynamics	Angle between vertical axis and urethral axis (α angle), posterior urethrovesical angle (ϐ angle), bladder neck mobility (densus diameter)	Transperineal B-mode ultrasound at rest and on straining (Valsalva), supine, bladder filled with 300 ml saline solution	Moderate	Low	No	SUI group had greater ϐ angles than controls at rest (121.6° ± 17.8° vs. 109.2° ± 7.5°, *p* = 0.01) and on straining (166.8° ± 33.3° vs. 123.1° ± 8.9°, *p* = 0.001). No significant difference in α angle at rest (17.5° ± 10.0° vs. 15.0° ± 6.7° for SUI group and controls respectively, *p* = 0.65). Significantly greater α angle during straining in SUI group compared to controls (51.0° ± 14.0° vs. 31.3° ± 11.7°, *p* = 0.001). Significantly greater densus diameter in SUI group (27.4 ± 7.2 mm) vs. control group (11.2 ± 4.4 mm, *p* = 0.001)	Matched for age. SUI group were older and had significantly higher parity and body mass than controls
Smith et al. (2007) [28] Australia	SUI: N = 16; age = 49.8 ± 12.0 years and BMI = 25.8 ± 4.5 kg/m^2^. Controls: N = 14; age = 52.5 ± 12.5 years and BMI = 25.1 ± 2.9 kg/m^2^. SUI confirmed with self-report on a severity scale	EMG amplitude (μV)	Surface EMG of abdominal muscles and PFM (Periform™ intravaginal probe) recorded prior to and after a loading perturbation in which a 1 kg weight was dropped 30 cm into a bucket held by the subject	High	High	No	SUI group had higher non-normalized PFM EMG than controls both prior to and during the postural response associated with unexpected loading. Non-normalized EMG of external obliques higher in SUI group than in controls	Matched for age, BMI, habitual physical activity level and parity
Snooks et al. (1985) [126] UK	SUI: N = 12 and age = 47 (22–68) years. Controls: *N* = 20 and age =? SUI confirmed through cystometrography	Perineal nerve and pudendal nerve terminal motor latency, single fiber density of external anal sphincter muscle and maximal voluntary contraction pressures in anal sphincter zone	Manometry and EMG evoked potentials	Moderate	Low	No	Perineal nerve terminal motor latency significantly slower in SUI group than controls (3.9 ± 0.8 ms vs. 2.0 ± 0.2 ms, *p* < 0.001). No other significant differences between groups	Matched for age and parity. BMI not reported
Stoker et al. (2003) [127] The Netherlands	SUI: *N* = 20. Controls: N = 20. Total age: 52 (40–65) years. SUI was defined based on clinical history, physical examination, and urodynamic examinations	Lesions of urethral support mechanism and lesions (defects and scars, thinning) of levator ani muscle	Endovaginal MRI	High	Low	Yes	Lesions more prevalent in SUI group than controls (*p* < 0.01). Lesions of urethral support system more prevalent in SUI group (9/20) than controls (2/20) and defects of levator ani present in SUI group (13/20) but not in controls. Median levator ani thickness in SUI group significantly lower than in controls (2.5 (0.9–4.1) mm vs. 3.9 (1.4–7) mm, *p* < 0.01)	Unadjusted. Matched for age. BMI and parity not reported
Takahashi et al. (2000) [128] Japan	SUI: *N* = 41 and age = 61.3 (45–79) years. Controls: N = 10 and age = 62.4 (51–77) years. SUI confirmed based on history, physical examination, roentgenological examinations, a 1-h pad test and urodynamic evaluations	Duration and amplitude of motor unit potentials (MUP) and number of phases	EMG of striated urethral sphincter	High	Low	No	Compared to controls MUP in the SUI group had shorter duration (*p* = 0.001), lower amplitude (*p* = 0.001) and larger number of phases (*p* = 0.002)	Unadjusted. No significant difference in age between groups and both groups had histories of vaginal deliveries. BMI and parity not reported
Thompson et al. (2006) [24] Australia	SUI: *N* = 30; age = 46.4 ± 5 years and BMI = 24 ± 4 kg/m^2^. Nulliparous controls: N = 30; age = 39.7 ± 9 years and BMI = 23 ± 3 kg/m^2^. Parous controls: N = 30; age = 41.8 ± 5 years and BMI = 22 ± 3 kg/m^2^. SUI determined with an interview and graded with the Incontinence Severity Index (ISI)	Bladder neck depression during PFM contraction, bladder neck elevation during maximum effort voluntary PFM contraction. PFM strength (Oxford scale), vaginal squeeze pressure (cm H_2_0) and PFM endurance time (seconds)	Transperineal ultrasound imaging (supine, participants asked to drink 500 ml water), manual muscle testing, and Peritron perineometer	Moderate	Low	Yes	Control women significantly stronger on manual muscle testing and perineometry and had greater PFM endurance than women with SUI. No difference in amount of bladder neck movement during PFM contraction between patients with SUI and continent women, but there was a trend toward greater amount of bladder neck elevation during maximum effort voluntary PFM contraction in the continent women than the incontinent women. Bladder neck depression during PFM contraction more common in women with SUI than controls	Unadjusted. SUI group significantly older than nulliparous but not parous controls. No significant differences in BMI between groups. No difference in mean parity between parous controls and SUI group
Varma et al. (1988) [129] UK	SUI: N = 28 and age = 41 (24–63) years. Controls: N = 28 and age = 44 (18–75) years. SUI confirmed with clinical examination and conventional videocystometry	Electrosensitivity of dorsal nerve of clitoris and of urethral mucosa, reflex latency measurements (dorsal nerve to external anal sphincter, dorsal nerve to urethral sphincter, urethral mucosa to external anal sphincter), motor unit potential amplitude and duration of external anal sphincter, voluntary sphincter contraction pressure	Urethral catheterization, perineal electrical stimulation, anorectal manometry	Moderate	Low	No	Three different reflex latency measurements were longer in SUI group than controls (*p* < 0.01). Dorsal nerve and urethral mucosal electrosensitivity lower in SUI group than controls (*p* < 0.01). SUI had significantly longer motor unit potential duration of external anal sphincter compared to controls (*p* < 0.01). Voluntary sphincter contraction pressures significantly lower in SUI group than controls (*p* < 0.02)	Unadjusted. Matched for age and parity. BMI not reported
Verelst and Leivseth (2007) [130] Norway	SUI: N = 21; age = 46 (27–54) years and BMI = 26 (20–43) kg/m^2^. Controls: *N* = 24; age = 40 (30–49) years and BMI = 22 (19–26) kg/m^2^. SUI classification unknown	Passive and active forces generated by the pelvic floor. Stiffness was defined as change in force divided by change in diameter of the dynamometer arms	Custom Intravaginal dynamometer	High	Low	No	Passive forces did not differ between the groups (*p* = 0.65). Compared to controls the SUI group had significantly lower active force development (0.092 ± 0.055 N/kg vs. 0.161 ± 0.115 N/kg, *p* = 0.03) and lower stiffness (0.008 ± 0.007 ΔN/Δ mm vs. 0.014 ± 0.010 ΔN/Δ mm, *p* = 0.006)	Adjusted for age, parity and body weight. SUI group were older and had higher BMI and parity than controls
Weidner et al. (2000) [131] USA	SUI: N = 9 and age = 54.3 (35–75) years. Controls: N = 15 and age = 28.7 (20–49) years. SUI confirmed urodynamically	Inference pattern: number of turns per second and mean signal amplitude (μV); contraction strength (0–9)	Concentric needle electrode EMG of levator ani and external anal sphincter	High	Low	No	Greater number of turns/s in SUI group than controls for levator ani (*p* = 0.07), greater number of turns/s in control group than SUI group for external anal sphincter (*p* = 0.02). Levator ani strength 9 (5–9) in controls and 5 (2–7) in SUI group	Unadjusted. SUI group significantly older than controls. BMI not reported. Controls were nulliparous and SUI group parous
Weil et al. (1993) [132] The Netherlands	SUI: N = 22 and age = 48 (28–67) years. Controls: *N* = 33 and age = 38 (27–66) years. SUI confirmed with urodynamics	Bladder neck position (distance from bladder neck to apex of pubic bone and to point where line from apex of pubic bone intersects with line perpendicular to probe), “rotational angle” (angle between a line drawn through bladder neck parallel to probe and a line through apex of pubic bone), posterior uretrovesical angle (PUVA)	Urodynamic evaluation, 2D B-mode transperineal ultrasound at rest and during coughing, supine, bladder filled to at least 200 ml	High	Low	No	In SUI group bladder neck was lower and in a more dorsal position at rest and during coughing than in controls (*p* < 0.001). “Rotational angle” larger in SUI group than controls both at rest (36° ± 18° vs. 18° ± 18°, *p* < 0.001) and during coughing (46° ± 27° vs. 23° ± 19°, *p* < 0.001). No significant difference in PUVA at rest or during coughing between groups (*p* > 0.05). No significant differences between groups in change from rest to coughing except in change in distance from bladder neck to apex of pubic bone (*p* < 0.001)	Unadjusted. Not matched for age or parity. SUI group mean age older than controls. BMI not reported
Wlaźlak et al. (2018) [133] Germany	SUI: *N* = 210; age = 49 (22–82) years. Controls: *N* = 447; age = 50 (19–86) years. BMI: 27 (16–41) kg/m^2^ in both groups. SUI confirmed through positive cough test	Urethral funneling length, urethral funneling width, length of the urethra	Transvaginal sonography was performed with women in supine and with a full bladder to assess urethral length and urethral funneling during maximal Valsalva maneuver	Moderate	Low	No	In all patients with SUI, urethral funneling and urine flow were visible, and urethral funneling length was > 50% of total urethral length. In 83.7% of women without SUI urethral funneling was absent. In the remaining 16.3% of women without SUI, funneling was visible but its relative length was < 50% of the total urethral length	Unadjusted. No difference between controls and SUI group in distribution of age, BMI, number and mode of deliveries
Yang and Huang (2002) [134] China	SUI: *N* = 764 and age = 49 ± 11 years. Controls: *N* = 36 and age = 48 ± 12 years. SUI confirmation not reported	Funneling of bladder neck, rotational angle of bladder neck, bladder wall thickness and bladder neck–symphyseal angle	Ultrasonographic cystourethrography at rest and during straining (Valsalva), supine, comfortably full bladder and then emptied	High	Low	No	Funneling of bladder neck in 35% of SUI group and 0% of controls. SUI group had greater bladder neck-symphyseal angle compared to controls at rest (97° ± 23° vs. 81° ± 15°, *p* < 0.01) and during straining (152° ± 34° vs. 113° ± 27°, *p* < 0.01). Greater rotational angle of bladder neck in SUI group than controls (56° ± 29° vs. 30° ± 20°, *p* < 0.01). Greater bladder wall thickness in SUI group than controls (6.0 ± 2.4 mm vs. 4.9 ± 2.1 mm, *p* < 0.05)	Unadjusted. Similar age and parity of groups. BMI not reported
Yang et al. (2013) [135] Taiwan	SUI: *N* = 208 and age = 50.9 ± 10.4 years. Controls: *N* = 126 and age = 50.7 ± 8.6 years. SUI was confirmed through symptoms and urodynamic evidence	Presence of reflex PFM contraction (inward clitoral motion during coughing, superior-anterior lift of the anorectal angle during coughing)	Transperineal ultrasound with the probe placed over the introitus, supine, bladder comfortably full then emptied	Moderate	Low	No	No difference in inward clitoral reflex motion or in anorectal reflex motion between SUI group and controls	Unadjusted. No significant difference in age, parity, BMI and percentage of postmenopausal women between SUI group and controls
Yang et al. (2019) [32] China	SUI: *N* = 241; 32.0 ± 4.3 years and postpartum BMI = 23.1 ± 2.9 kg/m^2^. Controls: *N* = 1139 age = 31.1 ± 4.3 years and postpartum BMI = 22.6 ± 3.1 kg/m^2^. Postpartum women. SUI confirmed with urodynamics	Oxford grading system for palpation. EMG amplitude (μV)	Digital palpation and EMG using pear-shaped vaginal probe during phasic, tonic and endurance contractions	High	High	No	No difference in PFM palpation. Lower EMG amplitudes of SUI group than controls at rest and during contraction (*p* < 0.05). Significant, moderate correlation between PFM strength and EMG in both groups (r = 0.467–0.545, *p* < 0.001)	Not adjusted. SUI group significantly older than controls, but not a meaningful difference and likely due to large sample size. Similar BMI and parity between groups
Zhang et al. (2013) [14] China	SUI: *N* = 50 and age = 51.26 ± 8.40 years. Controls: N = 50 and age = 50.76 ± 14.14 years. SUI was confirmed urodynamically	Maximum flow rate, maximum urethral pressure, maximum urethral closure pressure; functional urethral length. Bladder neck funneling length movement of the urethrovesical junction, angle of rotation of the urethrovesical junction, posterior urethrovesical angle (PUV)	2D transperineal B-mode ultrasound at rest and during straining, supine, bladder filled to 250 ml and urodynamic measurements including uroflowmetry, filling and voiding cystometry and urethral pressure profilometry	Moderate	Low	No	Rotation angle and movement of urethrovesical junction, funneling of the bladder neck and PUV angle at rest and during straining were greater in SUI group than in controls (*p* < 0.001). Maximum flow rate significantly higher in the SUI group than in the control group (*p* < 0.001). Maximum urethral pressure, maximum urethral closure pressure, and functional urethral length significantly lower in SUI group than in controls (*p* < 0.05)	Unadjusted. Matched for age. BMI and parity not reported
Zhao et al. (2020) [136] China	SUI: *N* = 40; 51.4 ± 10.5 years and BMI = 23.7 ± 1.9 kg/m^2^. Controls: N = 40 age = 50.8 ± 9.8 years and BMI = 22.6 ± 2.9 kg/m^2^. SUI confirmed with interview and positive cough test or urodynamics	Bladder neck descent (BND), urethral rotation angle (URA), and retrovesical angle (RVA). Maximally caudal organ positions (of bladder neck, uterus and rectal ampulla) relative to the pubic symphysis. Young’s modulus and shear wave velocity	Transperineal ultrasound with supersonic shear wave imaging, supine after bladder emptying	High	Low	Yes	Significantly greater BND in SUI group (25.4 ± 7.8 mm) vs. controls (18.0 ± 4.1 mm, *p* = 0.01) and less lowest bladder position on maximum Valsalva relative to symphysis in SUI group (0.1 ± 5.8 mm) vs. controls (8.9 ± 4.5 mm, *p* < 0.01). Less mean and maximum Young’s Modulus and shear wave velocity in SUI vs. controls (*p* = 0.001–*p* = 0.018)	Unadjusted. No significant difference in age, BMI or parity between groups

BMI: body mass index; EMG: electromyography; ICIQ-UI SF: International Consultation on Incontinence Questionnaire- Urinary Incontinence Short Form; MRI: magnetic resonance imaging; MUCP: maximal urethral closure pressure PFM: pelvic floor muscle; SUI: stress urinary incontinence

## Footnotes

1 These supportive tissues are often referred to as the “pubo-urethral ligaments,” although they do not insert exclusively into the pubic bone or attach directly to the urethra [139].

2 Rotational angle of the bladder neck was defined as the angle between lines from the symphysis pubis to the bladder neck [14, 90], as the difference between two angles formed by the intersection of a line from the symphysis pubis to the urethra and the sympysis pubis to the bladder neck [87] at rest and during straining, respectively.

3 Sliding was described as the difference between the urethra-bladder neck distance (the distance from the crossing point of the symphysis axis with the urethra to the bladder neck) at rest compared to on straining [87].
