# Correction to: The pathophysiology of stress urinary incontinence: a systematic review and meta-analysis

**DOI:** 10.1007/s00192-021-04794-y

**Published:** 2021-04-17

**Authors:** Kobra Falah-Hassani, Joanna Reeves, Rahman Shiri, Duane Hickling, Linda McLean

**Affiliations:** 1 Faculty of Health Sciences, University of Ottawa, Ottawa, Canada; 2 Finnish Institute of Occupational Health, Helsinki, Finland; 3 Ottawa Hospital Research Institute, Ottawa, Canada; 4 Department of Surgery, Division of Urology, The Ottawa Hospital, Ottawa, Canada

**Correction to: International Urogynecology Journal (2021) 32:501–552**

10.1007/s00192-020-04622-9

The article “The pathophysiology of stress urinary incontinence: a systematic review and meta-analysis”, written by Kobra Falah-Hassani, Joanna Reeves, Rahman Shiri, Duane Hickling, and Linda McLean, was originally published Online First without Open Access. After publication in volume 32, issue 3, page 501–552 the author decided to opt for Open Choice and to make the article an Open Access publication. Therefore, the copyright of the article has been changed to © The Author(s) 2021 and the article is forthwith distributed under the terms of the Creative Commons Attribution 4.0 International License, which permits use, sharing, adaptation, distribution and reproduction in any medium or format, as long as you give appropriate credit to the original author(s) and the source, provide a link to the Creative Commons license, and indicate if changes were made. The images or other third party material in this article are included in the article’s Creative Commons license, unless indicated otherwise in a credit line to the material. If material is not included in the article’s Creative Commons license and your intended use is not permitted by statutory regulation or exceeds the permitted use, you will need to obtain permission directly from the copyright holder. To view a copy of this license, visit http://creativecommons.org/licenses/by/4.0/.

The original article has been corrected.

